# Enhancing cow manure composting via staged inoculation of functional microbial consortia

**DOI:** 10.3389/frmbi.2026.1855343

**Published:** 2026-08-11

**Authors:** Huanyao Li, Weimin Zeng, Jin Huang, Shiyong Tan

**Affiliations:** 1 Central South University, School of Minerals Processing and Bioengineering, Changsha, Hunan, China; 2 Key Laboratory of Biometallurgy, Ministry of Education, Changsha, Hunan, China; 3 Hunan Tevos Ecological Technology Co., Ltd, Changsha, Hunan, China

**Keywords:** aerobic composting, humic substances, microbial community, microbial consortia, pot experiments, staged inoculation

## Abstract

Aerobic composting of cattle manure is often limited by slow humification and long duration. This study evaluated a staged inoculation strategy using phase-specific microbial consortia to enhance composting efficiency. Cow manure and rice straw were composted under four treatments: no inoculant (W), staged commercial EM inoculant (EM), single initial composite inoculant (TF), and staged targeted consortia (YF). The YF treatment achieved the longest thermophilic phase (11 days, peak 62.87 °C), the highest humic substances (122.02 g/kg) and humic acid (92.32 g/kg), and the highest total nitrogen (19.20 g/kg) with a seed germination index of 90.63%. Pot experiments using the resulting composts on pakchoi showed that the YF-derived organic fertilizer (YFP) significantly improved soil available nitrogen, phosphorus, and potassium, increased plant height and root length, enhanced chlorophyll content, and reduced superoxide dismutase activity compared to other treatments. Staged inoculation with targeted consortia effectively modulated microbial community succession, promoting lignocellulose degradation and humus synthesis. These findings demonstrate that phase-synchronized microbial management is a promising strategy to accelerate composting, improve product maturity, and enhance agronomic performance, supporting sustainable agricultural waste recycling.

## 1 Introduction

Meat consumption in China has been on the rise year by year, with increasing demand driving up livestock production and scale, leading to rapid development in the livestock industry (Li et al., 2022; Pan et al., 2023). The rapid growth of the livestock industry has resulted in a significant increase in the production of animal waste, with China generating approximately 4 billion tons of poultry and livestock manure annually (Yang et al., 2023). China is also an agricultural powerhouse, producing approximately 870 million tons of crop straw annually (Zhou et al., 2024). The improper disposal of livestock manure and crop straw poses new challenges to the global ecological environment. Currently, there are still widespread instances of crop straw being piled up in fields or burned in concentrated areas. This not only severely pollutes the environment but also results in significant resource waste. Livestock manure, particularly cattle manure, contains abundant nutrients such as nitrogen and phosphorus, as well as harmful substances like antibiotics, parasite eggs, and harmful bacteria (Sun et al., 2024; Nauanova et al., 2025). If improperly handled or utilized, it not only wastes organic materials but also causes environmental pollution and may even threaten human health (Meng et al., 2022; Aboutayeb et al., 2025). Therefore, developing efficient and sustainable waste management strategies is not only an agronomic necessity but also an environmental imperative, contributing to the circular economy and sustainable agricultural development.

Traditional methods for handling livestock waste include feed conversion, incineration, anaerobic digestion, and aerobic composting (Wang et al., 2024). Livestock manure is a potential high-quality organic solid waste with high production volume, high organic matter content, and ease of degradation, making it suitable for composting. Aerobic composting is a complex reaction process driven by microorganisms, involving the degradation of large amounts of organic matter and the continuous transformation of various chemical substances (Wang et al., 2023). Based on temperature changes during the composting process, aerobic composting can be divided into four stages: heating phase (mesophilic phase), thermophilic phase, cooling phase, and maturation phase (Luo et al., 2024; Qv et al., 2024). During the composting process, part of the organic matter is decomposed into CO_2_ and H_2_O, while mineralization occurs, and another part of the organic matter is resynthesized to produce complex and stable humus (Lu et al., 2018). Humification is an important process in aerobic composting, and the quantity and quality of humus directly determine the quality of the compost product. Humus not only improves the physical, chemical, and biological properties of soil but also promotes plant growth, reduces soil erosion, and remediates soil pollution (Zhang et al., 2023; Han et al., 2025).

The addition of functional microorganisms is an important method to accelerate the decomposition and maturation of organic matter and shorten the composting time (Duan et al., 2020). Microbial activity plays a key role in the biological transformation of organic substrates during the composting process. Bacteria are the most widely distributed and abundant microorganisms in composting, and current research on bacterial community succession is the most extensive (Zhang et al., 2023). Bacteria play a significant role in the degradation of organic matter, proteins, lipids, cellulose, and lignin during the composting process (Wang et al., 2023). Additionally, fungi play an important role in the composting process, being the primary microbial group responsible for degrading lignocellulosic materials. Research indicates that Ascomycota and Basidiomycota can secrete various cellulase and hemicellulase enzymes, making them the primary fungal decomposers in livestock manure and agricultural waste composting (He et al., 2022). In the initial stage of aerobic composting of cattle manure, inoculating a composite microbial inoculant containing *Bacillus amyloliquefaciens* and *Aspergillus glaucus* can effectively accelerate temperature rise in the compost pile, prolong the thermophilic phase, expedite the maturation process, and concurrently reduce methane (CH_4_) emissions—contributing to rapid composting maturation and pathogen inactivation (Impraim et al., 2020). Adding carbon-based microbial inoculants at the beginning of composting can accelerate the decomposition process, increase microbial diversity, accelerate the succession of microbial communities, and the decomposition and metabolism of materials, and promote the decomposition process (Tran et al., 2012). Duan et al. (2020) inoculated *Bacillus subtilis* into cattle manure-straw compost, which increased the seed germination index of the compost, extended the thermophilic phase of composting, increased humus content, accelerated compost maturation, and improved compost quality. During different stages of composting, the dominant microbial communities vary and play distinct roles. In the mesophilic phase, the primary microbes include mesophilic bacteria, fungi, and cellulose-decomposing microorganisms (such as thermophiles, fungi, and actinomycetes). During the thermophilic phase, cellulose-decomposing microorganisms (*thermophiles* and *actinomycetes*) dominate. Compared with bacteria, fungi have weaker tolerance to high temperature, so they mainly play a role in the heating and cooling stages of composting (Luo et al., 2024). Zhou et al. (2015) conducted composting by inoculating three different inoculants at three distinct stages. Inoculant A was added before fermentation, Inoculant B after the thermophilic phase, and Inoculant C on day 30 of fermentation. The results demonstrated that these inoculants significantly enhanced both the compost temperature and the lignocellulose degradation rate. However, researches about staged Inoculation to enhance composting were still limited.

Employing dairy manure and rice straw substrates, this study implemented staged inoculation of designed microbial consortia during composting to assess microbiome-mediated process enhancement. The objectives were: (1) To screen and isolate dominant functional microorganisms from different phases (mesophilic, thermophilic, cooling, maturation) of cow manure composting and formulate them into phase-specific microbial consortia; (2) To systematically evaluate and compare the effects of staged inoculation (YF) against a single-dose inoculation (TF), a commercial staged inoculant (EM), and a non-inoculated control (W) on key physicochemical parameters, humification processes, and overall compost maturity; and (3) To elucidate the interrelationships between the evolution of critical environmental factors (e.g., temperature, pH, TOC, TN) and the dynamics of bacterial and fungal communities throughout the composting process. Through this approach, we aim to validate a novel strategy of staged inoculation with targeted indigenous consortia, providing fundamental insights into microbiome-mediated composting enhancement and a practical framework for improving the efficiency and sustainability of agricultural waste management.

## 2 Materials and methods

### 2.1 Compost raw materials and pot experiment materials

Cow manure and rice straw were used as the raw materials for composting. The cow manure was sourced from a farm in Anhua County (Hunan Province, China), and the rice straw was sourced from an agricultural base in Anhua County, Hunan Province. The rice straw was chopped into pieces approximately 2 cm in length. The added microbial inoculant were EM(Effective Microorganisms)microbial inoculant and homemade microbial inoculant. EM microbial inoculants are commonly available organic fertilizer microbial inoculant (2×10^10^ CFU/g) purchased from Junde biotechnology company in Shandong Province, China. EM inoculant mainly includes *Bacillus*, *Saccharomyces*, *Rhodopseudomonas*, *Acetobacter*, *Lactobacillus*, *Streptomyces*, and *Trichoderma*. The physical and chemical parameters of the raw materials for cow manure composting are shown in **Table 1**.

**Table 1:** Basic physical and chemical properties of cow manure and rice straw.

Parameters	Cow manure	Rice straw
MC%	19.86	9.84
OM (g/kg)	660.79	877.14
TOC (g/kg)	235.16	483.84
TN (g/kg)	14.1	8.6
C/N	16.7	56.3

The soil used in the pot experiment was collected from a hill in Anhua County, Hunan Province. The livestock waste organic fertilizers consisted of four types of cow manure organic fertilizers obtained through composting. The cow manure organic fertilizers were air-dried, ground, and passed through a 2 mm sieve. Pakchoi (also known as Chinese cabbage) was selected as the test crop. Because, as a common leafy vegetable, it is easy to cultivate, has a short growth cycle, and can be marketed in just over a month, making it an ideal test crop. The experimental pots had a diameter of 16 cm and a height of 19 cm. The soil organic matter content (SOM)was 16.51 g/kg, and the contents of available nitrogen (AN), available phosphorus (AP), and available potassium (AK) were 90.08, 126.50, and 68.56 mg/kg, respectively.

### 2.2 Targeted microbial inoculant screening and species identification

Phase-defined compost samples served as source material for isolating indigenous microorganisms, subsequently formulated into phase-adapted inoculant consortia. Briefly, a mixture of 10 kg cow manure and 5 kg rice straw was prepared uniformly, added laboratory-prepared composting microorganisms, and adjust the initial moisture content of the material to approximately 60%. Composting is conducted in a fermentation tank, which is cylindrical in shape, with a diameter of 30 cm, a height of 80 cm, and a volume of 50 L (**Supplementary Figure 5**). The fermentation equipment has three temperature probes at the top, middle, and bottom on the front, and three sampling ports at the top, middle, and bottom on the back. The central axis of the fermentation tank is connected to a mechanical device with stirrers at the top, middle, and bottom layers. The bottom of the fermentation tank is made of porous material connected to an air pump, with aeration adjusted to 0.45 L·min^-^¹·kg^-^¹. Samples were collected from three sampling ports of the compost, with 60 g taken from each port. The samples were then mixed thoroughly and used for subsequent analysis. Samples (180 g) were collected on days 0, 2, 7, 14, 21, and 30. Samples collected at the mesophilic, thermophilic, cooling, and maturation stages were serially diluted to obtain dilutions of 10^-^¹, 10^-^³, 10^-5^, 10^-7^, and 10^-9^. Aliquots of the dilutions were spread onto solid media and incubated. Subsequently, the streak plate method was used to isolate and purify bacteria to obtain pure cultures. Genomic DNA was extracted from the purified inoculants, sequenced, and the resulting sequences were finally uploaded to the NCBI database for taxonomy annotation (Query Coverage >98%, Per. Ident >99.1%). The composite microbial inoculants for each stage were constructed based on the following principles: (1) relative abundance of microorganisms at each stage; and (2) functional representativeness of the screened and purchased strains. Strains were blended at a 1:1 ratio, and the microbial inoculants obtained from each stage were directly used as microbial inoculants for subsequent experiments at the same stage. (Detailed information about the source material for isolating indigenous microorganisms is provided in the **Supplementary Material**).

### 2.3 Composting enhancement experiment design

The experiment was set up with four composting groups: the control group (W) without any inoculant, the EM group with EM inoculant added in stages, the TF group with a composite inoculant added in a single dose at the beginning of composting, and the YF group with staged addition of different compost-enhancing inoculant. In the YF treatment, phase-specific consortia were added at the mesophilic, thermophilic, cooling, and maturation stages according to the microbial functional requirements at each stage, ensuring optimal performance of each strain under appropriate temperature and substrate conditions. This strategy prevented the death of mesophilic microorganisms during the thermophilic phase or the inactivation of lignin-degrading microorganisms due to a lack of precursors. In the TF treatment, all strains from the YF treatment were pre-mixed into a single composite inoculant and added only once at the start of composting. The EM inoculant had a viable cell count of 2 × 10¹^0^ CFU/g, while the phase-specific targeted inoculant had a viable cell count of 9 × 10^9^ CFU/g. According to the manufacturer’s instructions for the EM inoculant, its application rate was 0.4% (w/w). To achieve a comparable viable cell load, the phase-specific inoculant was applied at 0.9% (w/w). For the TF treatment group, all isolated microorganisms were blended into a single custom inoculant and applied once at composting initiation (0.9% w/w total). Corn-cob powder (cellulose 65%, hemicellulose 30%, on a dry weight basis. The particle size ranged from 100 to 200 mesh, providing a specific surface area conducive to microbial adsorption and colonization.)equivalent to YF’s cumulative supplement was added at corresponding phases thereafter. The blank control group received phase-matched cornmeal supplements equivalent to other treatments’ carrier inputs. The targeted microbial inoculant or corn-cob powder was added on days 0, 5, 14, and 22 during the composting.

### 2.4 Physical and chemical analysis of compost samples

Moisture content (MC) was determined using the constant weight method. Specifically, 5 g of fresh sample was placed in a 105 °C oven (DHG-9140A, China) and dried to constant weight. The moisture content was calculated as the weight loss. Total organic carbon (TOC) content of air-dried samples was determined using combustion oxidation-non-dispersive infrared absorption spectroscopy. 5 g of air-dried samples were mixed with 1 mol L^−1^ KCl at a ratio of 1:20 (w/v), shaking for 20 min, filtering the supernatant, and then using a TOC analyzer (SHIMAZU TOC-LCPH, Japan) to measure the total organic carbon (TOC) content. 0.5 g of air-dried sample was digested with H_2_SO_4_ and HClO_4_ for total nitrogen (TN) analysis, following the standard method (Wu et al., 2025). Total organic carbon (TOC)/total nitrogen (TN) is defined as C/N. 10 g of fresh sample were mixed with distilled water at a 1:10 (w/v) ratio and stirred thoroughly, after which the supernatant was collected. The electrical conductivity (EC) and pH of the supernatant were measured using a conductivity meter (DDS-307A, China) and a pH meter (PHS-3C, China), respectively. Then, 5 ml of the supernatant was placed in a petri dish containing filter paper. Twenty soybean seeds were evenly distributed on the filter paper and incubated at 28 °C in the dark for 72 hours. The seed germination rate and root length were recorded, using distilled water as the control. The germination index (GI) was calculated using Equation 1 (Duan et al., 2020):

(1) $$GI\left(\%\right)=\frac{Seed\;Germination\;Rate\times Mean\;Root\;Length\;of\;Treatment}{Seed\;Germination\;Rate\times Mean\;Root\;length\;of\;Control}\times 100$$

Air-dried sample (5 g) was taken and mixed with the extraction solution (0.1 M Na_4_P_20_·10H_2_O + 0.1 M NaOH) at a ratio of 1:10 (w/v), then stirred for 24 hours. Then the supernatant was filtered via 0.45 μm Millipore membrane, the filtrate was the humic substances (HS) solution. Thereafter, the filtrate was further separated to obtain humic acid (HA) and fulvic acid (FA). Adjusting the pH of the filtrate to 1 with 6 M HCl and standing for 12 h at 4 °C. Then centrifuged the acidic solution obtained in the previous step at 10,000 rpm for 10 min, then the supernatant was filtered via 0.45 μm Millipore membrane, and the filtrate was the FA solution, and the precipitate was dissolved by 0.05 M NaHCO_3_ to form the HA solution. The concentration of HS, HA and FA was measured by organic carbon analyzer (Wang et al., 2021). Humification parameters are calculated based on TOC, HS, HA and FA using Equations 2–5 (Wu et al., 2025):

(2) Humification ratio(HR)=(HS/TOC)×100

(3) Humification index(HI)=(HA/TOC)×100

(4) Percentage of humic acids(PHA)=(HA/HS)×100

(5) Degree of polymerization(DP)=(HA/FA)×100

Fresh sample (10 g) was mixed with distilled water at a ratio of 1:10 (w/v), shaked at 25 °C for 24 hours, then centrifuged to collect the supernatant. The supernatant was filtered through a 0.45 μm membrane filter to obtain dissolved organic matter (DOM). The sample was scanned using a molecular fluorescence spectrometer (F-700, Japan). The excitation wavelength (Ex) was set from 200 nm to 500 nm, the emission wavelength (Em) from 220 nm to 600 nm, the scan increment to 5 nm, and the scan speed to 2400 nm min^-^¹. Distilled water was used as the background value to obtain the three-dimensional fluorescence excitation-emission matrix (EEM) map of the sample.

### 2.5 Pot experiment design and physicochemical parameter analysis

Five treatments were established: organic fertilizer treatment from Group W (WP), organic fertilizer treatment from Group TF (TFP), organic fertilizer treatment from Group EM (EMP), organic fertilizer treatment from Group YF (YFP), and a no-fertilizer treatment (CK). Each treatment had three replicates. Each pot was filled with 2.5 kg of soil, and based on preliminary experiments, the organic fertilizer application rate was set at 3% (W/W). The experiment was conducted in a constant temperature and humidity incubator (HCQ-1000, China) with a light intensity of 4000 LX, a 12 h light/12 h dark photoperiod, a relative humidity of 60%–80%, and the temperature controlled at 20–30 °C. Irrigation was carried out every five days. Ten pakchoi seeds were sown per pot and covered with a thin layer of soil. Three days after seedling emergence, healthy seedlings with consistent growth were selected and retained in each pot, leaving four seedlings per pot with spacing of approximately 8 cm. Plants were harvested 45 days after sowing.

Soil samples were collected using a multi-point mixed sampling method on days 1, 15, and 45, with 50 g of surface soil (0–20 cm) collected each time. Fresh samples were used for direct measurement of pH and electrical conductivity (EC). Collected soil samples were air-dried, ground, and passed through a 0.15 mm sieve for analysis of soil organic matter (SOM), available nitrogen (AN), available phosphorus (AP), and available potassium (AK). The methods for determining pH, EC, and soil organic matter content are the same as those described in Section 2.4. According to the method of Guo et al. (2025), soil available nitrogen content was determined by the alkali hydrolysis diffusion method, soil available phosphorus content was measured by the sodium bicarbonate-molybdenum antimony colorimetric method, and soil available potassium content was determined using a flame photometer. Plant samples of pakchoi were collected at 15 and 45 days of growth for measurement of plant height, root length, chlorophyll content, and superoxide dismutase (SOD) activity. Plant height and root length were measured using a ruler graduated in millimeters. Chlorophyll content in the samples was determined using a BC0990 Plant Chlorophyll Content Assay Kit (Solarbio, Beijing) according to the manufacturer’s instructions. SOD activity was measured using a Superoxide Dismutase (SOD) Assay Kit from Nanjing Jiancheng.

### 2.6 Extraction of genomic DNA from compost samples

Samples from Days 2, 7, 21, and 30 corresponded to the mesophilic, thermophilic, cooling, and maturation composting phases, respectively, with triplicate replicates per sample. Genomic DNA was extracted from each compost sample using the DNeasy PowerSoil kit (QIAGEN, Dusseldorf, Germany) according to the manufacturer’s instructions. The quality of the extracted DNA was verified using 1% agarose gel electrophoresis. The V3–V4 region of the bacterial 16S rRNA gene was amplified using the primer pair 515F (5′-GTGCCAGCMGCCGCGG-3′) and 907R (5′-CCGTCAATTCMTTTRAGTTT-3′), while the fungal ITS region was amplified using the primer pair ITS5 (5′-GGAAGTAAAAGTCGTAACAAGG-3′) and ITS4 (5′-TCCTCCGCTTATTGATATGC-3′). The PCR products were verified by 2% agarose gel electrophoresis and purified using the AMPure XT beads kit (Beckman, USA). The purified samples were sent to LC-Bio Technology Co. Ltd. (Hangzhou, China) for high-throughput sequencing on their OmicStudio platform. Raw sequencing data were deposited in the NCBI Sequence Read Archive (SRA) under accession number PRJNA1467625.

### 2.7 Statistical analysis and visualization

All analyses were repeated three times to obtain the average value. Data were analyzed using one-way analysis of variance (ANOVA) in Excel to determine significant differences between treatments, with p< 0.05 indicating significant differences. Data visualization was performed using Origin 2024. Alpha diversity analysis was conducted using the Shannon index, with data calculated using Mothur v1.30.1 and visualized using R software v.3.4.4. Beta diversity distance matrices were calculated using Qiime software, and non-metric multidimensional scaling (NMDS) analysis was performed using R software v.3.4.4 based on bray-curtis distance. Redundancy analysis (RDA) was used to investigate the relationships between environmental factors and microbial communities, with data analysis conducted using CANOCO 5.0.

## 3 Results and discussion

### 3.1 Sources of targeted inoculant

During the composting process, the succession of microbial communities leads to stage-specific differences in the primary substances they decompose (Liu et al., 2022). During the mesophilic phase, microorganisms primarily decompose easily decomposable organic matter such as soluble starch, sugars, and proteins. Microorganisms decompose residual complex carbohydrates, macromolecular fats, and proteins during the high-temperature phase, progressively degrading complex components such as cellulose, hemicellulose, and lignin. Subsequently, in the cooling phase, microorganisms specifically target lignin and other recalcitrant substances. By the maturation phase, microorganisms further decompose residual difficult-to-degrade organic substances, promoting compost stability (Luo et al., 2024). In this study, 44 pure-culture strains with decomposition activity were screened from compost samples (as shown in **Supplementary Table 2**). These screened strains aligned well with the key phase dominant microbiota identified in the previous microbial community species composition analysis. During the mesophilic phase, the dominant bacteria were *Providencia stuartli*, *Klebsiella* sp. *HE1 strai*n, and *Brevundimonas* sp. *RTB strain*. During the thermophilic phase, the dominant bacteria are *Halomonas* sp. *NIMMe9 strain*, *Bacillus subtilis D32 strain*, and *Bacillus subtilis FX-8 strain*. The dominant bacteria during the cooling period were H*alomonas* sp. *NIMMe9, Alcanivorax* sp. *YDC2-H 16S, Rhodococcus rhodochrous strain JS-24*, and *Bacillus velezensis*. The dominant bacteria during the maturation period were *Luteibacter anthropi strain GP5* and *Halomonas* sp. *BJGMM-B48*. To enhance the efficiency of composting, some strains that are important for composting were obtained from the China General Microbiological Culture Collection Center (CGMCC) and the China Center of Industrial Culture Collection (CICC) and supplemented into our composite inoculants. The phase-specific microbial inoculants are listed in **Supplementary Table 1**.

### 3.2 Changes in physical and chemical parameters

**Figure 1:**
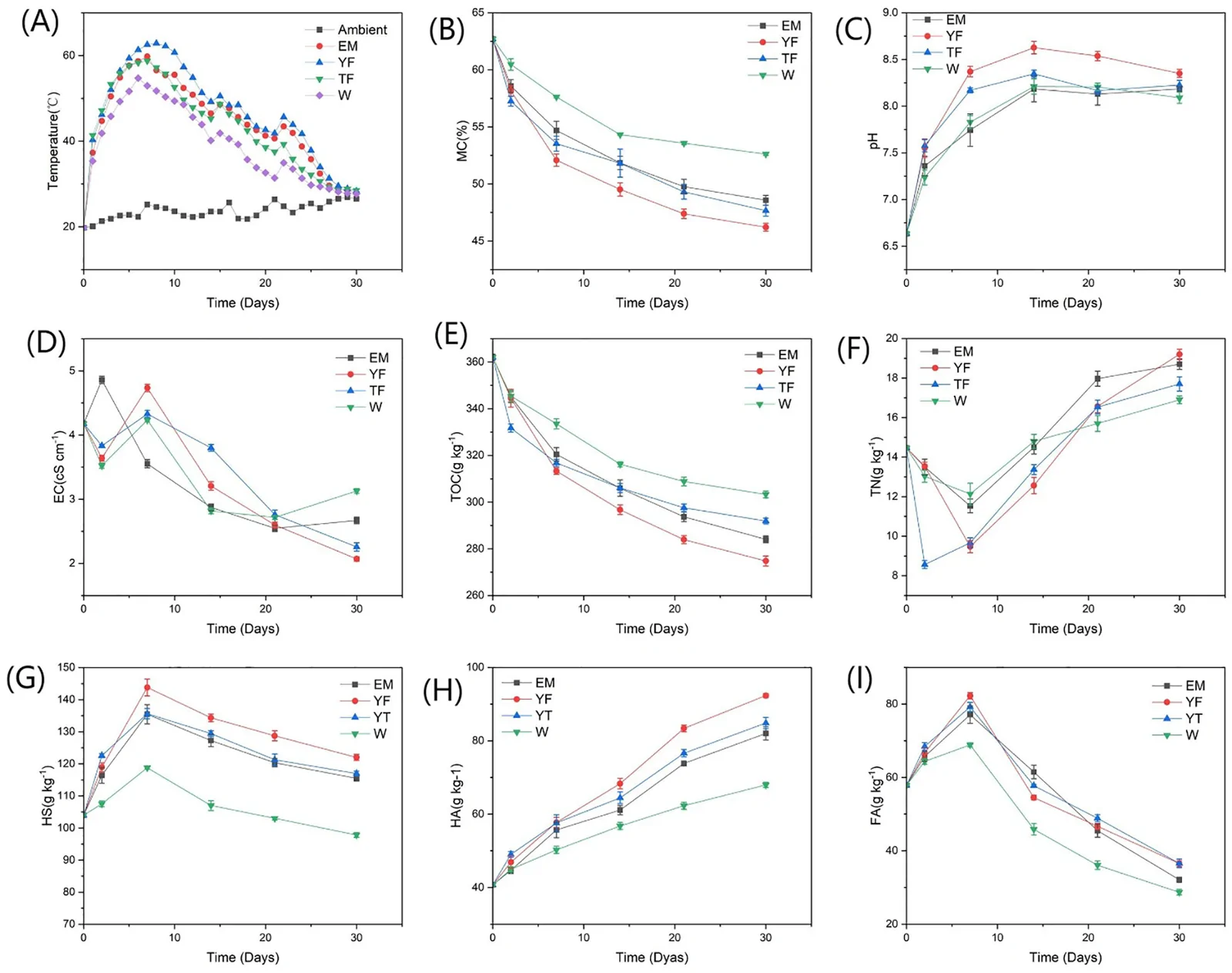
Changes in composting process temperature **(A)**, moisture content **(B)**, pH **(C)**, electrical conductivity **(D)**, total organic carbon **(E)**, total nitrogen **(F)**, humus **(G)**, humic acid **(H)**, and fulvic acid **(I)** after adding composting microbial inoculants. (“W “indicates no inoculant added, “EM” indicates EM inoculant added in stages, “TF” indicates composite inoculant added once at the start of composting, “YF” indicates targeted inoculant added in stages).

Temperature is a key parameter in composting, as high temperatures can eliminate insect eggs and pathogens (Chang et al., 2019). The temperature changes during the composting process are shown in **Figure 1A**. A rapid rise in initial composting temperature was observed in all four treatments, with the temperatures reaching above 50 °C on the third day in the EM, TF, and YF treatments, while the temperatures reaching above 50 °C on the fourth day in the control group. The thermophilic phase (≥50 °C) was significantly prolonged in the YF group (11 days), exceeding durations in EM (10 days) and TF (8 days) groups, with a peak temperature reaching to 62.87 °C in the YF group, meeting the Chinese industry standard (NY/T 3442-2019). This result was consistent with previous studies that adding microorganisms to the composting system accelerates organic matter decomposition and releases more heat (Wang et al., 2023).

Moisture content in the compost pile affects microbial growth and regulates temperature. Excessively low moisture content inhibits microbial metabolism, slowing down the composting process. When moisture content is too high, it restricts ventilation and creates an anaerobic environment, delaying the composting process (Aboutayeb et al., 2025). The moisture content of all treatment groups showed a decreasing trend, influenced by evaporation, ventilation, and organic matter decomposition (Liu et al., 2021). During the composting mesophilic phase and thermophilic phase, microbial decomposition of organic matter releases a large amount of heat, causing rapid moisture loss in the pile (as shown in **Figure 1B**). At the end of composting, the moisture content of each treatment group was 48.58 ± 0.43% (EM), 46.21 ± 0.35% (YF), 47.66 ± 0.48% (TF), and 52.62 ± 0.11% (W), with the lowest moisture content observed in the YF group. This may be due to the higher temperature and longer thermophilic period in the YF treatment, which promoted water evaporation.

Microorganisms thrive under neutral or slightly alkaline conditions. During composting, the pH tends to rise, which may be due to the decomposition of protein-based organic matter by microorganisms, resulting in the production of large amounts of ammonia nitrogen and an increase in pH (Zhao et al., 2019). By day 30 of composting (as shown in **Figure 1C**), the pH of all treatment groups was between 8.1 and 8.5, meeting the requirement of “Technical Specifications for Sanitary Treatment of Livestock and Poultry Manure” (GB/T36195-2018) in China.

EC reflects the concentration of soluble salts in the compost pile, with higher concentrations indicating that the compost product is less suitable for application. During the early stages of composting (the first seven days), microbial decomposition of organic matter and rapid water evaporation caused the EC value to rise (as shown in **Figure 1D**). As temperature decreases, EC values show a downward trend, which may be due to increased cation exchange capacity and precipitation of different ions (Zhou et al., 2018). At the end of composting, the EC values of all treatments were below 4 mS cm^-^¹, indicating low salt hazard risk for the compost product (Li et al., 2022).

TOC exists in the form of organic matter and reflects compost quality. During composting, TOC content tends to decrease (Cao et al., 2023). In the first seven days of composting, rapid mineralization of organic matter under microbial action causes a rapid decrease in TOC content (as shown in **Figure 1E**). During composting, part of the carbon component of TOC is converted into CO_2_, while the remaining part forms humus (Yamada and Kawase, 2006). As composting progresses, microbial decomposition products generate humic acid-like substances (Zhou et al., 2014). The TOC content at the end of composting for EM, YF, TF, and W treatments was 284.07 g/kg, 274.81 g/kg, 291.93 g/kg, and 303.29 g/kg, respectively, representing decreases of 21.54%, 24.10%, 19.37%, and 16.23% compared to the beginning of composting. A greater decrease in TOC content was observed in the EM and YF groups, which may be attributed to the staged addition of microbial inoculants to promote organic matter decomposition.

Nitrogen exists in various forms during composting, with organic nitrogen being the primary form in compost. During composting, organic nitrogen undergoes mineralization and decomposition, and nitrogen is prone to form NH_3_ and other nitrogen-containing gases during transformation, leading to nitrogen loss (Wang et al., 2023). In the early stages of composting (as shown in **Figure 1F**), nitrogen content decreased across all treatments, as organic nitrogen was converted into NH_4_^+^-N under microbial action, resulting in volatilization or loss with moisture. After exposure to high temperatures, nitrogen content increases. This occurs due to mass reduction from organic matter decomposition during composting, which concentrates nitrogen content on a mass basis (Zhang et al., 2023). At the end of the composting process, the total nitrogen content in the YF treatment group was the highest (19.20 g/kg), indicating that the YF treatment can reduce nitrogen loss in compost.

### 3.3 Changes in humus during the composting process

In aerobic composting, the primary processes are the mineralization and humification of organic matter. Humification is the conversion of organic matter into humus, which is a key step in carbon sequestration during composting. Humus primarily consists of humic acid (HA) and fulvic acid (FA), with FA exhibiting higher activity than HA (Lu et al., 2024). In all treatment groups, humus content rapidly increased during the early stages of composting and then gradually decreased, which was similar to the study of Zhang et al. (2023). This is because, during the early stages of composting, microorganisms decompose easily degradable organic matter in the compost pile to form structurally stable humus. After decomposing the easily degradable organic matter, microorganisms utilize the humus, leading to a decrease in humus content. At the end of composting (as shown in **Figure 1G**), the HS content of the EM, YF, TF, and W treatments was 115.47 g/kg, 122.02 g/kg, 116.99 g/kg, and 97.83 g/kg, respectively. Compared to the control group, the HS content of EM, YF, and TF treatments significantly (*p* < 0.05) increased by 18.02%, 24.72%, and 19.57%, respectively. During composting, the content of HA gradually increases. As shown in **Figures 1H**, **I**. At the beginning of composting, the content of HA is lower than that of FA. As composting progresses, the fulvic acid content increases first and then gradually decreases (Li et al., 2022). During composting, microorganisms can utilize FA for metabolism and convert it into more stable HA, thereby enhancing the humification degree of the compost pile (Zhou et al., 2023). At the end of composting, the HA content of the EM, YF, TF, and W treatments was 82.02 g/kg, 92.32 g/kg, 84.84 g/kg, and 67.98 g/kg, respectively. Compared with W, the HA content of EM, YF, and TF was significantly (*p* < 0.05) increased by 20.65%, 35.81%, and 24.80%, respectively. Xu et al. (2022) added microbial inoculants to cattle manure composting, resulting in increases in HS and HA content of 11.03% and 27.58%, respectively, at the end of the composting process. This indicates that the YF treatment can significantly increase HA content and promote HS formation.

**Figure 2:**
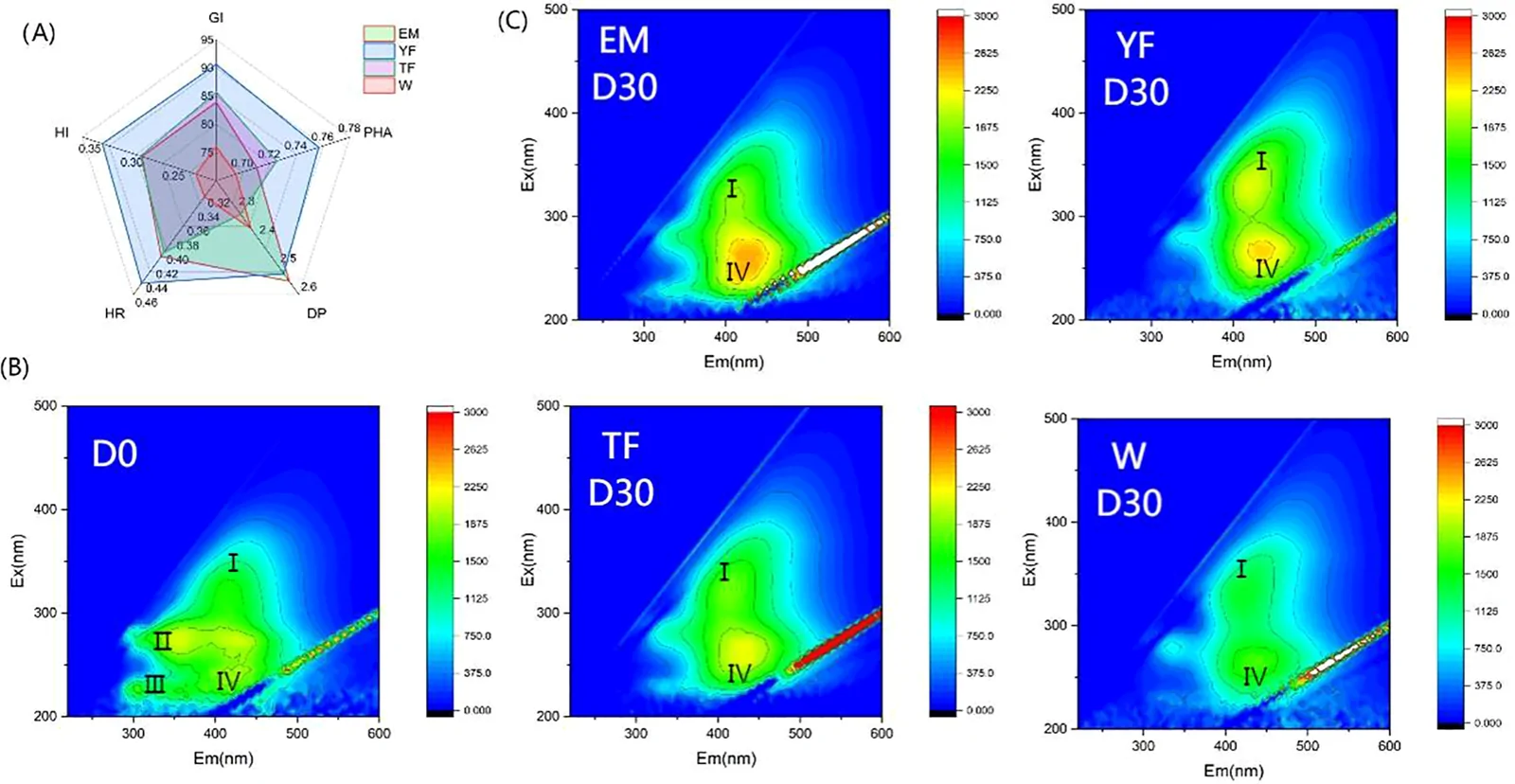
Humification parameters of compost products **(A)**, Excitation-Emission Matrix (EEM) spectra of dissolved organic matter in initial samples **(B)** and final samples **(C)**. (“W “indicates no inoculant added, “EM” indicates EM inoculant added in stages, “TF” indicates composite inoculant added once at the start of composting, “YF” indicates targeted inoculant added in stages).

During the composting process, microbial metabolism primarily occurs in the water-soluble phase. Studying the soluble organic matter in compost can help assess its maturity. Compost materials have a complex structure, and EEM can detect changes in the fluorescence intensity or peak position of DOM during the composting process (Droussi et al., 2009). At the start of composting (as shown in **Figure 2B**), four peaks appeared in the sample spectrum: Ex/Em 375/450 nm (Peak I, associated with humic acid), 270/340 nm (Peak II, associated with soluble microbial byproducts), 230/340 nm (Peak III, associated with tyrosine-like proteins), and 250/440 nm (Peak IV, associated with FA) (Li et al., 2022). By the end of composting, Peaks II and III had almost disappeared, leaving only Peaks I and IV, as microorganisms decomposed and metabolized proteins and soluble substances during the composting process (Zhu et al., 2024a). In the 30-day samples (as shown in **Figure 2C**), the YF treatment exhibited the strongest fluorescence intensity for Peak I, indicating that the staged addition of microorganisms promotes the decomposition of substances and their conversion into more stable humic acid-like compounds (Kong et al., 2023). This shift in the fluorescence peaks reflects the increase in amino, alkoxy, hydroxy, carbonyl, and carboxyl groups during the composting process.

HR, HI, DP, and PHA are commonly used to assess compost maturity and humification levels (Wu et al., 2025). As shown in **Figure 2A**. Compared to the W treatment, the HR values of the EM, YF, and TF treatments were significantly (*p* < 0.05) increased by 26.21%, 37.65%, and 24.23%, respectively. HR represents HS/TOC, and the increase in HR values, especially in the YF treatment, indicates enhanced humification. The YF and EM treatment groups had higher HI, PHA, and DP values than the W group at the end of composting, indicating that staged addition of microbial inoculants can promote composting and improve maturity.

GI can reflect the strength of plant toxicity in the sample and is an economical and effective indicator for assessing compost maturity. Generally, a GI value greater than 80% indicates that the compost has achieved harmless effects (Kong et al., 2022). At the end of composting, the GI values for each group were 83.92 (EM), 90.63 (YF), 85.70 (TF), and 76.03 (W). The GI value for the W group was less than 80%, failing to meet the maturation standard. The GI values of the other three groups all exceeded 80%, with the YF group having the highest GI value.

### 3.4 Compost microbial community analysis

#### 3.4.1 Bacterial community analysis

**Figure 3:**
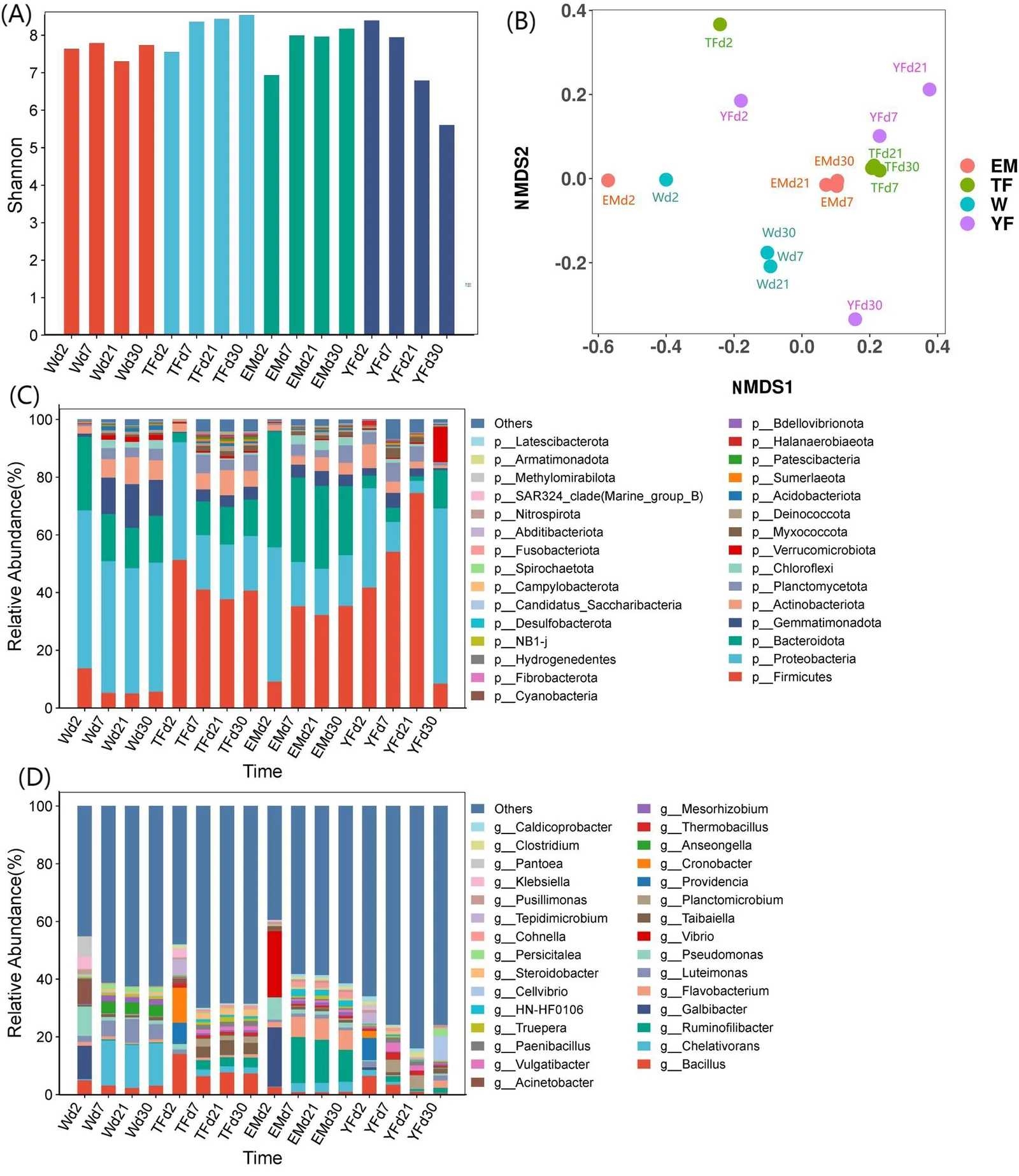
Changes in relative abundance and diversity of bacterial communities during composting: Shannon index **(A)**, NMDS analysis **(B)**, bacterial phylum level analysis **(C)**, bacterial genus level analysis **(D)**. (“W “indicates no inoculant added, “EM” indicates EM inoculant added in stages, “TF” indicates composite inoculant added once at the start of composting, “YF’ indicates targeted inoculant added in stages).

The Shannon index is employed to quantify species diversity within a community. A higher Shannon index indicates greater diversity. The bacterial Shannon index is presented in **Figure 3A**. The Shannon index for the TF and EM treatments exhibited an increasing trend, whereas the Shannon index for the YF treatment showed a decreasing trend. Research indicates that competition can occur between exogenous inoculants and indigenous microorganisms, as well as among indigenous microorganisms themselves (Zhao et al., 2022). Inoculation led to a reduction in bacterial community diversity (Zhu et al., 2023). This reduction in the YF treatment may be attributed to the staged addition of targeted inoculants. This targeted intervention impacted specific composting phases, thereby leading to a more specialized microbial community function. Non-metric Multidimensional Scaling (NMDS) based on the Bray-Curtis dissimilarity is shown in **Figure 3B**. NMDS analysis revealed distinct differences in bacterial community structure among the different treatment groups. Bacterial community dynamics in treatments W, TF, and EM followed similar trajectories. Specifically, samples clustered together after the thermophilic phase, consistent with findings reported in previous research (Li et al., 2025). In contrast, bacterial community structures in the YF treatment exhibited significant shifts across time points, while samples from different composting phases showed dispersed geometric distribution patterns. This indicates that the staged addition of our targeted inoculants can induce changes in the bacterial community composition during composting.

Changes in bacterial community composition at the phylum level during composting are illustrated in **Figure 3C**. Firmicutes and Proteobacteria typically dominate the composting process, although their relative abundances fluctuate over time. Proteobacteria often prevail initially, while Firmicutes increase during thermophilic and maturation phases. Bacteroidota, Gemmatimonadetes, and Actinomycetota are also common. Elevated *Firmicutes* abundances in treatments TF, EM, and YF indicated that microbial inoculants promoted enrichment of this phylum. As Firmicutes members contribute to thermotolerance, antibiotic resistance, and organic matter degradation (Fang et al., 2024), their predominance may explain the shorter thermophilic phase in the control group (W). This observation also explains the shorter thermophilic phase observed in the control group. At the bacterial genus level (**Figure 3D**), *Bacillus*, *Pseudomonas*, *Flavobacterium*, and *Luteimonas* were identified as common composting genera (Wei et al., 2018; Luo et al., 2024). In the control group (W), microorganisms with high relative abundance during the thermophilic phase were *Pseudomonas*, *Galbibacter*, and *Bacillus*. The predominant microbial types remained largely consistent post-thermophilic phase, primarily *Bacillus*, *Chelativorans*, *Luteimonas*, and *Anseongella*. For the treatment TF, *Bacillus* and *Cronobacter* exhibited high relative abundance in the thermophilic phase. Post-thermophilic dominant types showed minimal variation, dominated by *Bacillus*, *Taibaiella*, *Planctomicrobium*, and *Ruminofilibacter*. In the EM treatment group, thermophilic-phase high-abundance microorganisms included *Bacillus*, *Vibrio*, and *Galbibacter*, while *post*-*thermophilic* communities maintained similar dominant profiles featuring *Ruminofilibacter*, *Flavobacterium*, and *Chelativorans*. The dominant bacteria in the mesophilic phase of YF treatment were *Bacillus*, *Luteimonas* and *Cronobacter*, *Providencia*. The dominant bacteria in the thermophilic phase were *Bacillus*, *Planctomicrobium*, *Thermobacillus*, *Vulgatibacter*. The dominant bacteria in the cooling phase were *Bacillus*, *Planctomicrobium*, *Thermobacillus*, *Paenibacillus*. The dominant microorganisms in the maturation phase were *Flavobacterium*, *Luteimonas*, which aligned with the specific microbial taxa targeted for addition during those respective stages. Genus-level community succession demonstrated similarity across treatments W, TF, and YF, characterized by minimal changes in the relative abundance of dominant genera after the thermophilic phase. In contrast, different stages of composting in the YF treatment showed different composting advantage bacteria. *Luteimonas*, a common genus in composting, is closely associated with the aromatization and humification of dissolved organic matter. *Pseudomonas* degrades substances like proteins and cellulose (Fang et al., 2024). Genera such as *Bacillus* are intimately linked to organic matter degradation and humification (Jiang et al., 2023). *Flavobacterium* possesses genes for degrading cellulose and hemicellulose and can decompose most forms of these compounds (Tian et al., 2024). Consequently, the microbial community structure in YF treatment showed a predominance of efficient decomposers. Genus such as Bacillus and Thermobacillus are renowned for their thermotolerance and robust enzymatic machinery for degrading complex polysaccharides like cellulose and hemicellulose, which contributed to the prolonged thermophilic phase. Simultaneously, the prevalence of Pseudomonas and Flavobacterium, known for their metabolic versatility in decomposing proteins, lipids, and simple sugars, facilitated the rapid mineralization of organic matter and provided precursors for humus synthesis.

#### 3.4.2 Fungal community analysis

**Figure 4:**
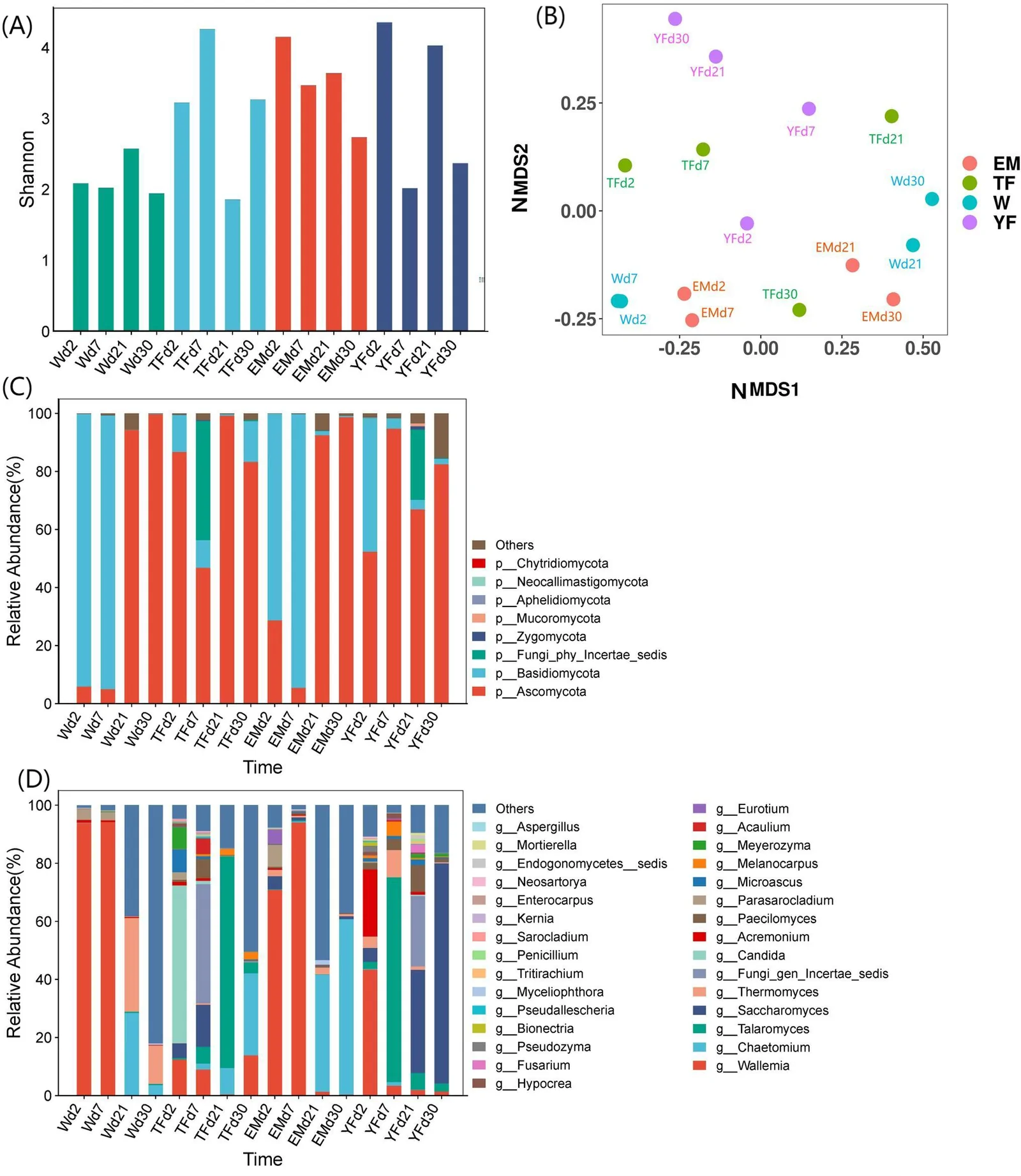
Changes in relative abundance and diversity of Fungal communities during composting: Shannon index **(A)**, NMDS analysis **(B)**, Fungal phylum level analysis **(C)**, Fungal genus level analysis **(D)**. (“W “indicates no inoculant added, “EM” indicates EM inoculant added in stages, “TF” indicates composite inoculant added once at the start of composting, “YF’ indicates targeted inoculant added in stages).

Changes in the fungal Shannon index are presented in **Figure 4A**. Compared to other treatments, the Shannon index in the control group (W) remained consistently low and stable throughout composting. The Shannon index in treatment TF exhibited multiphasic fluctuations—initially rising, then declining, before rising again. The Shannon index in treatments EM and YF displayed an initial decrease, followed by an increase, and then a subsequent decrease. Notably, during the maturation phase, the Shannon index of both the EM and YF treatment groups increased significantly. Overall, fungal α diversity typically exhibits a trend of being high in the early stages, fluctuating in the middle stages, and stabilizing in the mature stages. NMDS analysis based on Bray-Curtis dissimilarity (as shown in **Figure 4B**) showed that the samples in each treatment group did not form obvious clusters, which was consistent with the results of Wang et al. (2023). These results demonstrate that the staged addition of inoculants, particularly with targeted inoculants applied during specific composting phases, significantly influences fungal community succession.

Changes in fungal community composition at the phylum level during composting are shown in **Figure 4C**. The dominant fungal phyla were Ascomycota and Basidiomycota (collectively accounting for >90% of relative abundance), consistent with Wang’s findings (Wang et al., 2023). Basidiomycota were relatively more abundant during the early composting stage (days 0–7), while Ascomycota increased in relative abundance during the middle and late stages. At the genus level (as shown in **Figure 4D**), *Wallemia* and *Chaetomium* exhibited high relative abundances. *Chaetomium* promotes the degradation of organic matter throughout composting, generating small molecules such as amino acids, reducing sugars, and polyphenols, which serve as important precursors for HS formation (Yang et al., 2024). The fungal community in the control group (W) treatment was relatively simple, dominated mainly by *Wallemia* and *Chaetomiaceae*. In contrast, no fungi with a relative abundance higher than 80% were found in the TF and YF treatments, indicating that the addition of microbial inoculants resulted in a more complex fungal structure. During the initial stage of the YF treatment, *Wallemia* and *Chaetomium* were enriched, alongside the appearance of *Thermomyces* and *Saccharomyces*, corresponding to the fungi added at this stage. *Saccharomyces* became the dominant genus during the cooling and maturation phases of the YF treatment. Research by Zhu et al. indicates that *Saccharomycetaceae* are important in composting, capable of synthesizing polysaccharides and vitamins, thereby promoting stable coexistence within the microbial community through the establishment of metabolic exchange relationships (Zhu et al., 2024b). The high relative abundance of *Saccharomyces* in the YF treatment during the middle and late stages likely contributed to this stable microbial coexistence.

The succession of microbial communities directly drove the variations in physicochemical parameters observed in Section 3.2. The extended thermophilic phase and higher peak temperature in the YF group were concomitant with the early enrichment of thermophilic Bacillus and Thermobacillus, which accelerated organic matter decomposition and heat release. The significant reduction in TOC was strongly linked to the sustained activity of these efficient decomposers throughout the process. Furthermore, the remarkable increase in HA content and humification indices in the YF group can be attributed to the tailored succession of microbial communities: the early-stage decomposers provided substrates, while the mid- to late-stage populations, including Flavobacterium and Luteimonas, facilitated the polymerization and stabilization of these precursors into mature humic acids. This functional synchronization between inoculated consortia and physicochemical parameters underscores the efficacy of the staged inoculation strategy.

### 3.5 The association between microbial community and selected factors

**Figure 5:**
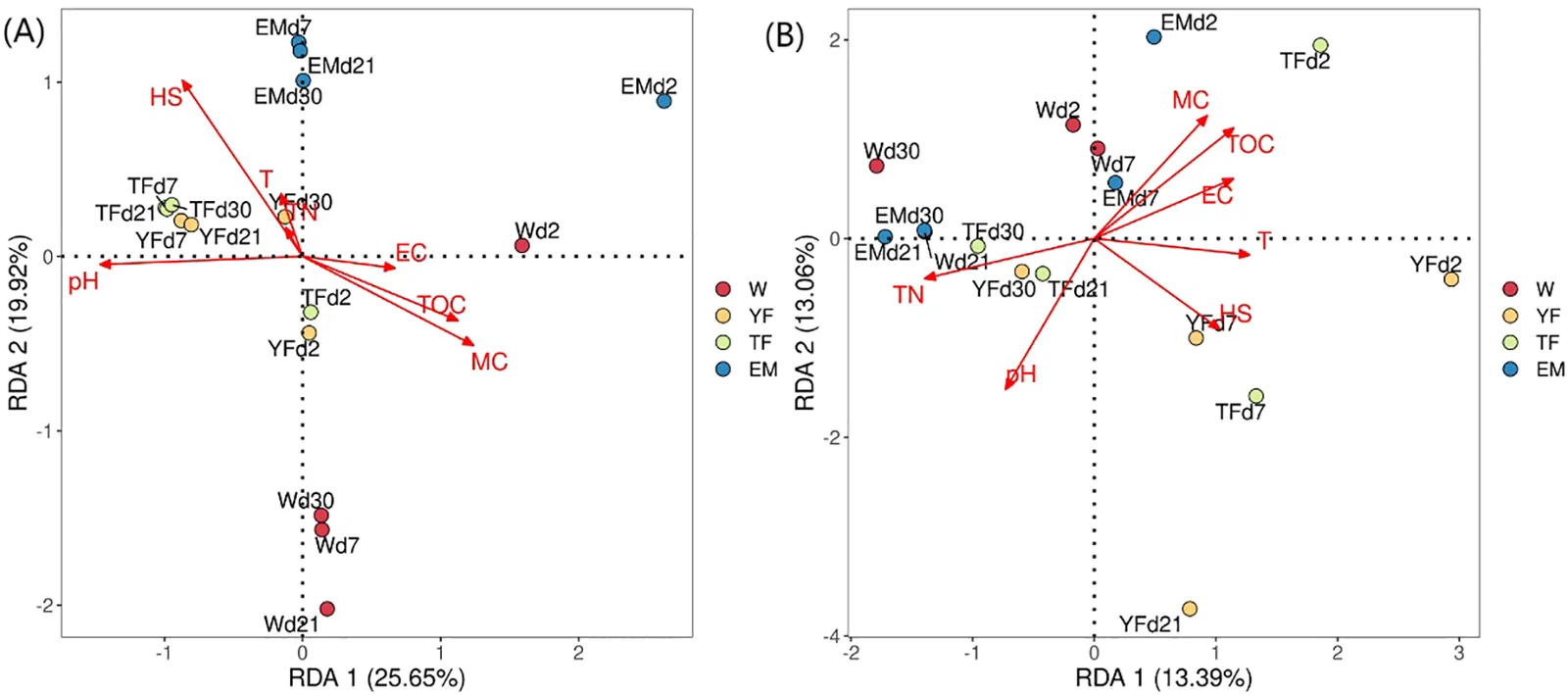
Redundancy analysis (RDA) of environmental factors and microbial communities: bacterial community **(A)**, fungal community **(B)**. (“W “indicates no inoculant added, “EM” indicates EM inoculant added in stages, “TF” indicates composite inoculant added once at the start of composting, “YF’ indicates laboratory-prepared composite inoculant added in stages).

Different physicochemical parameters during the composting process are associated with microbial community succession. Selected physicochemical parameters (TOC, TN, EC, pH, HS, MC, Temperature) and the top ten microbial genera by relative abundance were subjected to redundancy analysis (RDA). Redundancy analysis (RDA) revealed the explanatory power of environmental factors on microbial community structures (as shown in **Figure 5**). For bacterial communities (as shown in **Figure 5A**), the variance was primarily explained by pH, followed by MC, HS, TOC, EC, Temperature, and TN. For fungal communities (as shown in **Figure 5B**), the key explanatory factors were pH, TOC, MC, TN, EC, Temperature, and HS, in descending order of explanatory power. This ordination is visually represented in **Figure 5** by the length and direction of the arrows, with longer arrows indicating stronger influences. The influence of physicochemical parameters on bacteria and fungi exhibits different trends. Xiong et al. (2023) reported that during the composting process, the mechanisms driving nutrient transformation in bacteria and fungi are distinct, so the key factors driving the succession of bacterial and fungal communities may differ. Specifically, fungal succession is strongly regulated by TOC/TN dynamics due to their slower growth and carbon-dependent activity (Zhou et al., 2024), while pH universally influences microbial metabolism (Duan et al., 2020). Moisture content (MC) significantly modulates composting by affecting microbial diffusion and enzymatic reactions (Li et al., 2021; Kong et al., 2023). These findings corroborate the results of Wang et al. (2023), in which pH and TN shaped bacterial communities. Collectively, strategic modulation of key physicochemical parameters (pH, moisture content, total organic carbon) enables targeted restructuring of microbial communities, ultimately enhancing compost quality.

### 3.6 Physicochemical analysis of potted plant trials

**Figure 6:**
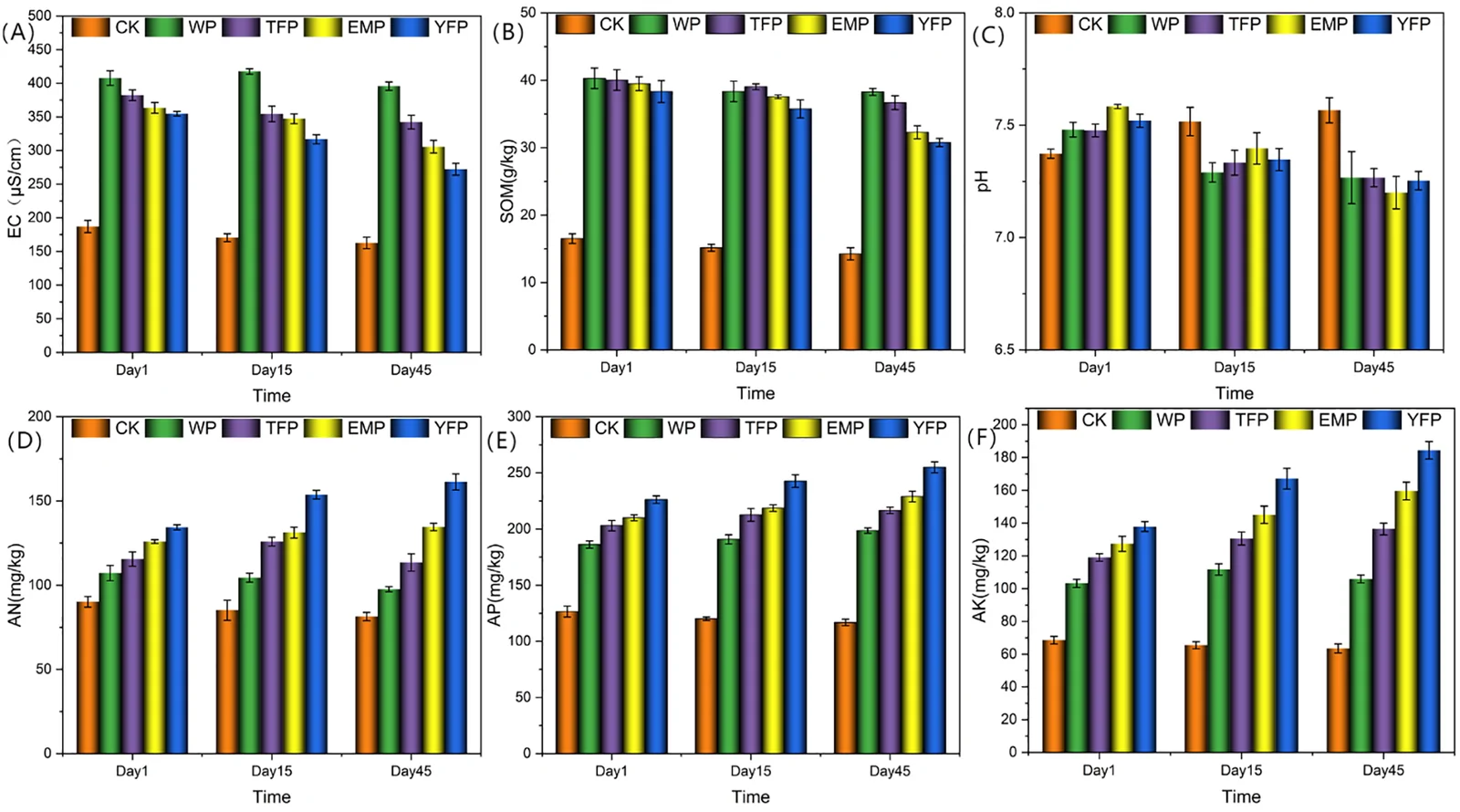
Soil physicochemical indicators under different fertilization treatments: EC **(A)**, pH **(B)**, Soil organic matter **(C)**, available nitrogen content **(D)**, available phosphorus content **(E)**, available potassium content **(F)**: organic fertilizer treatment from Group W (WP), organic fertilizer treatment from Group TF (TFP), organic fertilizer treatment from Group EM (EMP), organic fertilizer treatment from Group YF (YFP), and a no-fertilizer treatment (CK).

Electrical conductivity (EC) reflects soil salinity status and is an important indicator for assessing soil salinization risk. Organic fertilizers contain high salt content. As shown in **Figure 6A**, After application, EC values in all organic fertilizer treatments were significantly higher than in CK. On day 45, the EC value in the YFP treatment decreased to 272.19 μS/cm, representing a 23.3% reduction compared to day 1, which was significantly lower than that in WP (395.77 μS/cm), TFP (342.33 μS/cm), and EMP (305.76 μS/cm). The CK treatment had an EC value of 162.73 μS/cm, which remained significantly lower than all organic fertilizer treatments. In the YFP treatment, EC values continuously decreased over the 45-day period, with the largest reduction, indicating the lowest risk of salt accumulation. EC values in the WP treatment remained consistently high, possibly related to high soluble salt content in the organic fertilizer or low plant uptake efficiency. Generally, an EC value exceeding 1000 μS/cm indicates a salinization risk (Zhu et al., 2026). In this study, none of the treatments reached this threshold.

Soil pH affects soil enzyme activity, microbial reproduction, and plant growth (Zhao et al., 2022). As shown in **Figure 6B**, pH in the CK group increased slightly, while pH in the organic fertilizer treatment groups showed a decreasing trend. The application of organic fertilizers can increase soil pH, while green manure cultivation can decrease it. During organic fertilizer decomposition, small molecular organic acids may be produced, potentially reducing soil pH. The continuous increase in pH in the CK group may be related to the accumulation of calcium and magnesium ions or weakened nitrification (Zheng et al., 2026).

Soil organic matter (SOM) is a crucial indicator of soil fertility (Jiao et al., 2020). As shown in **Figure 6C**, SOM content in all treatments showed a decreasing trend during the experiment. The organic matter content in the YFP treatment decreased to 30.77 g/kg, a 19.7% reduction compared to day 1, which was the largest decrease among all organic fertilizer treatments. By comparison, WP decreased by 5.0%, TFP by 6.0%, and EMP by 18.2%. The decrease in YFP was similar to that in EMP but was significantly higher than those in WP and TFP (*P* < 0.05). SOM consumption was faster in the YFP treatment, indicating a higher degree of organic matter mineralization, strong microbial activity, and rapid nutrient release, which benefits plant uptake and utilization. SOM in CK decreased continuously, indicating continuous consumption of native soil organic matter without exogenous input.

Soil available nutrients are nutrients that plants can directly absorb and utilize. Phosphorus and potassium are important nutrient elements in soil. Soil available nitrogen (AN), available phosphorus (AP), and available potassium (AK) reflect the soil’s nutrient supply capacity for N, P, and K (Luo et al., 2025). They are exchangeable and water-soluble nutrients that can be exchanged from soil colloids or directly absorbed by plants. As shown in **Figures 6D–F**, on day 45, the available nitrogen (AN) content in the YFP treatment reached 161.29 mg/kg, which was 98.3% higher than that in CK (81.37 mg/kg), 65.2% higher than in WP, 42.1% higher than in TFP, and 19.8% higher than in EMP. The available phosphorus (AP) content in the YFP treatment reached 254.97 mg/kg, which was 118.2% higher than in CK (116.86 mg/kg), 28.4% higher than in WP, 17.7% higher than in TFP, and 11.4% higher than in EMP. On day 45, the available potassium (AK) content in the YFP treatment reached 184.40 mg/kg, which was 190.2% higher than in CK (63.53 mg/kg), 74.1% higher than in WP, 35.2% higher than in TFP, and 15.6% higher than in EMP. The application of cow manure organic fertilizer increased the contents of soil available nitrogen, available phosphorus, and available potassium, which is consistent with the findings of Guo et al. (2025). Among the groups, the YFP treatment exhibited higher available nutrient contents at all time points. AK in the CK treatment decreased continuously, reflecting the depletion of soil available nitrogen, phosphorus, and potassium due to crop uptake.

**Figure 7:**
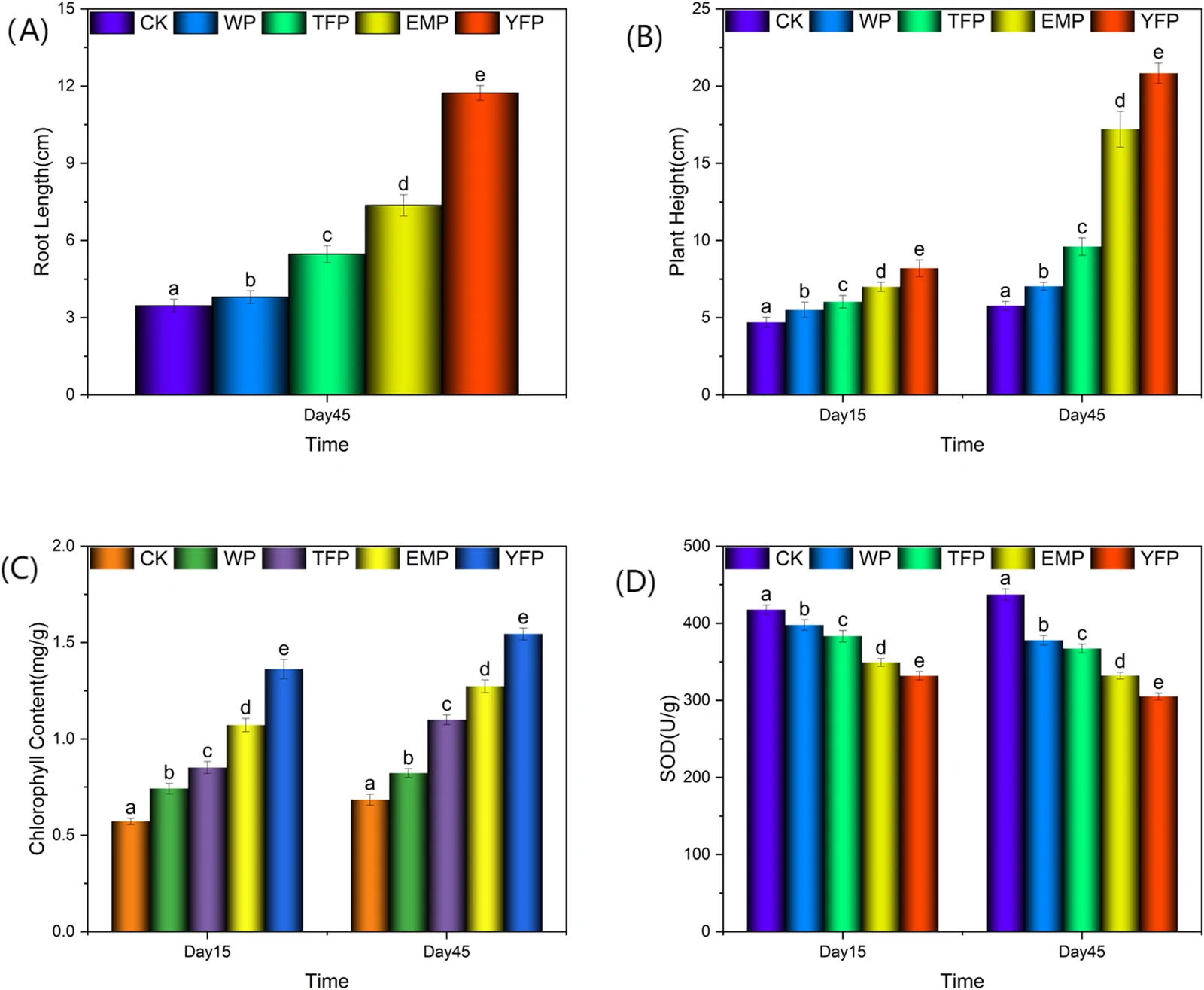
Pakchoi indicators under different fertilization treatments: root length **(A)**, plant height **(B)**, chlorophyll content **(C)**, SOD activity **(D)**: organic fertilizer treatment from Group W (WP), organic fertilizer treatment from Group TF (TFP), organic fertilizer treatment from Group EM (EMP), organic fertilizer treatment from Group YF (YFP), and a no-fertilizer treatment (CK). The lowercase letters (a, b, c, d, e) in represent the CK, WP, TFP, EMP, and YFP treatment groups, respectively.

Plant height and root length are intuitive indicators reflecting plant growth status (Jiang et al., 2024). As shown in **Figures 7A, B**, on day 45, the plant height in the YFP treatment reached 20.83 cm, which was 261.1% higher than in CK (5.77 cm), 196.3% higher than in WP, 117.0% higher than in TFP, and 21.1% higher than in EMP. The root length followed a trend similar to plant height, with the YFP treatment achieving a root length of 20.83 cm, representing a 261.1% increase compared to CK (5.77 cm). Both plant height and root length were highest in the YFP treatment, indicating a high match between nutrient supply and plant demand. The EMP treatment ranked second, while the TFP and WP treatments showed relatively weaker effects, possibly related to slower nutrient release rates from these organic fertilizers. The significant root development further enhanced nutrient uptake capacity, creating a positive feedback loop.

Chlorophyll content is an important indicator of photosynthetic capacity (Li et al., 2026). chlorophyll content in all groups continued to increase (**Figure 7C**). On day 45, the chlorophyll content in the YFP treatment reached 1.54 mg/g, which was 126.5% higher than in CK (0.68 mg/g), 87.8% higher than in WP, 40.0% higher than in TFP, and 21.3% higher than in EMP. The YFP treatment exhibited the highest chlorophyll content, which continued to rise, consistent with its highest available nitrogen content, indicating that sufficient nitrogen supply promoted chlorophyll synthesis and enhanced photosynthetic capacity. The CK treatment had the lowest chlorophyll content, reflecting the limitation of photosynthesis due to nitrogen deficiency. As shown in **Figure 7D**, on day 45, SOD activity in the YFP treatment decreased to 305.17 U/g, which was 30.2% lower than in CK, 19.2% lower than in WP, 16.9% lower than in TFP, and 8.1% lower than in EMP. The YFP treatment had the lowest SOD activity, indicating good plant growth, less accumulation of reactive oxygen species, and a lower level of oxidative stress. The CK treatment had the highest SOD activity, reflecting the activation of the plant’s antioxidant system under nutrient stress (Shah et al., 2025). This result is consistent with the optimal growth indicators observed in the YFP treatment, further validating its favorable nutrient supply and plant growth status.

## 4 Conclusions

In the YF group, targeted inoculations were added in stages to extend the high-temperature period (11 days), promoting the degradation and humification of organic matter. The HS and HA contents reached 122.02 g/kg and 92.32 g/kg, respectively, representing increases of 24.72% and 35.81% compared to the control group. Humification parameters (HR, HI, PHA, DP) and germination index (GI, 90.63%) collectively demonstrated optimal maturity with low salinity risk (EC< 4 mS/cm) in the treatment YF. While the one-time addition of microbial inoculant (TF) accelerated temperature rise, the high-temperature period was shorter than that of staged addition; the EM microbial agent had a weaker promoting effect on humification compared to the YF group. Staged addition of microbial agents enhanced cellulose/lignin degradation and humus synthesis by matching dominant microorganisms at each stage. The microbial diversity and abundance of functional bacteria in the YF group were significantly increased, driving the conversion of organic matter into stable humus and reducing nitrogen loss. Furthermore, pot experiments confirmed that the YF-derived organic fertilizer (YFP) outperformed other treatments in improving soil available nutrients (AN, AP, AK), promoting pakchoi growth (plant height, root length, chlorophyll), and alleviating oxidative stress (SOD activity), highlighting its superior agronomic efficacy. This study provides the first empirical framework for phase-synchronized microbial management via autochthonous consortia, revealing that temporal precision in community augmentation is critical for maximizing composting performance.

## Data Availability

The datasets presented in this study can be found in online repositories. The names of the repository/repositories and accession number(s) can be found in the article/**Supplementary Material**.
