# Supplementary material for: Enhancing cow manure composting via staged inoculation of functional microbial consortia
Source: Front Microbiomes. 2026 Aug 11;5:1855343. doi: 10.3389/frmbi.2026.1855343 (PMC13503620; doi:10.3389/frmbi.2026.1855343)
Supplement: Supplementary file 1 [file DataSheet1.pdf]

Supplementary Material

1 microbial screening

Luria-Bertani medium was prepared by dissolving Tryptone (10 g), yeast extract (5 g), and NaCl (10 g) in 1000 ml of distilled water. Beef extract peptone agar medium was formulated with Peptone (10 g), beef extract (3 g), and NaCl (5 g) dissolved in 1000 ml of distilled water. Potato Dextrose Broth medium was constituted by dissolving potato dextrose (5 g), peptone (10 g), glucose (15 g), and NaCl (5 g) in 1000 ml of distilled water. Actinophrys medium was prepared through dissolution of K_2HPO_4 (1 g), KNO_3 (1 g), $MgSO_4$ (0.5 g), $FeSO_4$ (0.01 g), NaCl (0.5 g), and soluble starch (20 g) in 1000 ml of distilled water. Solid media variants were obtained by adding 15 g of agar to each liquid medium. All media were autoclaved at 121°C for 20 minutes.

Bacterial suspensions were prepared by mixing samples from the warming-up, high-temperature, cooling-down, and maturation periods with sterile water. The suspensions were serially diluted with sterile water and plated on Luria-Bertani solid medium, beef extract peptone agar solid medium, and Actinophrys solid medium. After incubation, colonies were streaked repeatedly until purified isolates were obtained. Purified strains were cultured in liquid medium to logarithmic growth phase. Five-milliliter aliquots of bacterial culture were centrifuged at 10,000 g for 4 minutes to pellet cells. Genomic DNA was extracted from purified strains using a bacterial DNA extraction kit (Tiangen Biotech, Beijing) according to manufacturer protocols. DNA quality was assessed using a NanoDrop ND-1000 spectrophotometer and 1% agarose gel electrophoresis. The 16S rRNA gene was amplified by PCR with universal primers 27F (5′ -AGAGTTTGATCCTGGCTCAG-3′) and 1429R (5′ -GGTTACCTTGTTACGACTT-3′). PCR products were verified by 0.8% agarose gel electrophoresis prior to commercial sequencing (Qingke Biotechnology, Beijing). Sequences were submitted to the NCBI database for similarity analysis. (Query Coverage >98%, Per. Ident >99.1%).

Based on the Spearman correlation analysis (Figure 1), multiple coexisting synergistic interaction modules were formed within the composting microbial community. Most of the genera corresponding to the microorganisms isolated from compost (e.g., *Bacillus*, *Providencia*, *Klebsiella*, *Halocella*, *Caldicoprobacter*, etc.) appeared in the correlation matrix. In terms of strong positive correlations ($\rho \geq 0.8$), *Halocella* exhibited extremely strong positive correlations with multiple genera, including *M55D21_unclassified*, *Caldicoprobacter*, *Bacillales_unclassified*, *Symbiobacterium*, *Limnochordia_unclassified*, and *Limnochordaceae_unclassified*. *Bacillus* showed positive correlations with *MBA03_unclassified*, *Bacillaceae_unclassified*, and *Thermobacillus*. *Providencia* displayed strong positive correlations with *Klebsiella*, *Psychrobacter*, and *Cronobacter*. *Klebsiella* also showed high positive correlations with *Psychrobacter* and *Cronobacter*. *Psychrobacter* exhibited an extremely strong positive correlation with *Cronobacter*. *Thermobacillus* showed strong positive correlations with *Limnochordaceae_unclassified* and S0134*_terrestrial_group_unclassified*. *Limnochorda* exhibited strong positive correlations with *Halocella* and *Bacillales_unclassified*. In terms of moderate positive correlations ($0.6 \leq \rho < 0.8$), *Bacillus* was positively correlated with *Caldicoprobacter* and *Symbiobacterium*; *Providencia* with *Acinetobacter*; *Klebsiella* with *Acinetobacter*; and *Psychrobacter* with *Acinetobacter*. These positive correlations further confirmed the extensive synergistic interactions among different functional genera during composting, particularly within the core thermophilic degradation group and among the warming-stage microbial communities, which jointly promoted the decomposition and transformation of organic matter through mechanisms such as metabolic

complementarity and cross-feeding. Regarding negative correlations (ρ ≤ -0.8), *Providencia* exhibited strong negative correlations with *Novibacillus* and *Chelativorans*. *Klebsiella* also showed negative correlations with *Novibacillus*, indicating resource competition or metabolic inhibition among these microbial groups. Overall, the composting microbial network displayed a distinct modular structure, with positive correlation modules centered on *Halocella* and *Bacillus* dominating the decomposition of materials during the thermophilic and cooling stages.

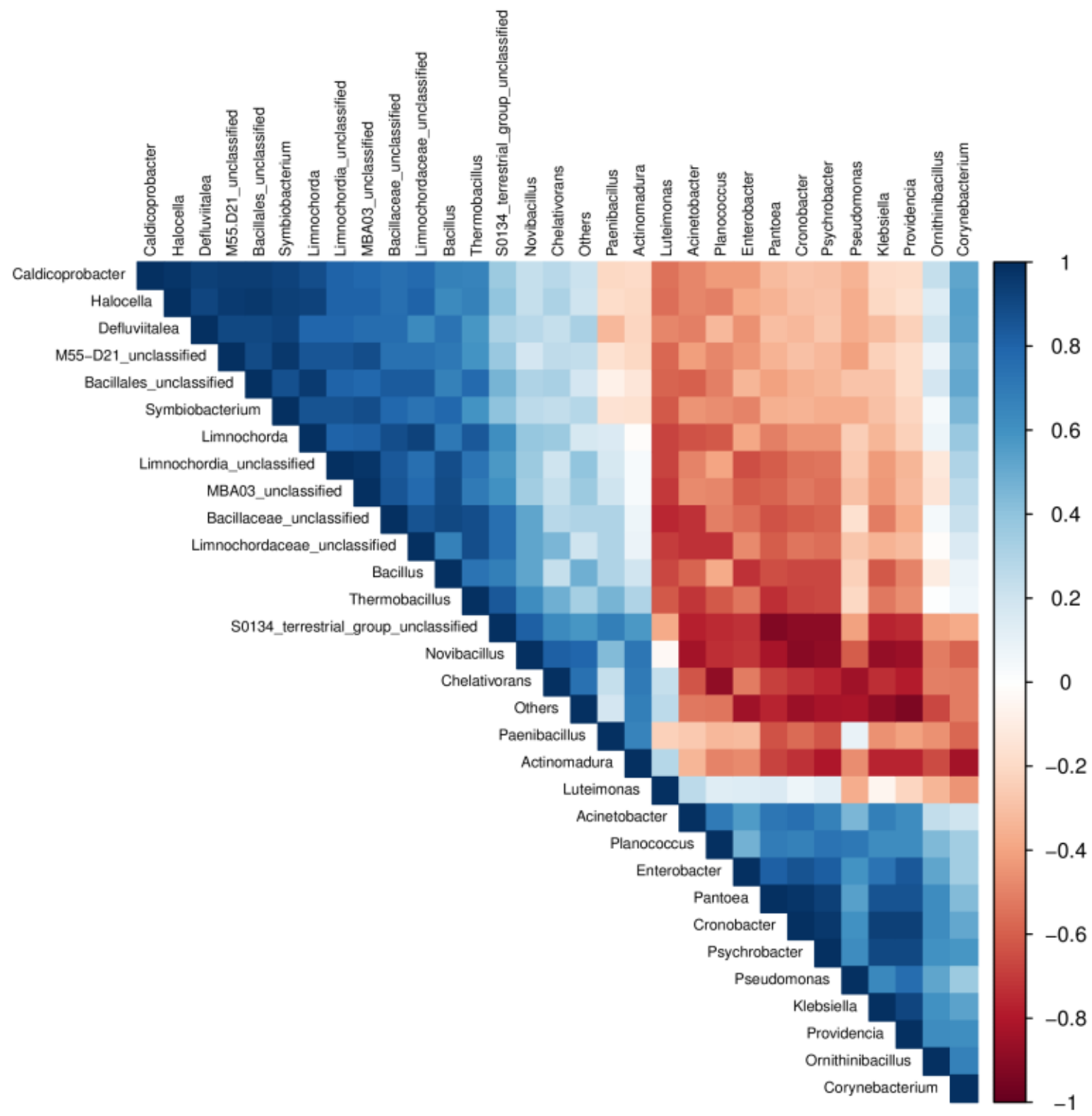

Supplementary Figure 1 Correlation diagram of composting microorganisms

2 Composting Effect Evaluation

Temperature reflects changes in the composting process and microbial activity, making it one of the most important indicators in composting(Zhao et al., 2016). The temperature changes during the composting process are shown in **Fig.S2**. On the third day of composting, the temperature rose to 52.3°C, reaching a peak of 61.5℃ on the sixth day. The thermophilic phase lasted for 10 days (>50°C), meeting the requirements of China's “Technical Specifications for Sanitary Treatment of Livestock and Poultry Manure” (NY/T 3442-2019). After the thermophilic phase, the temperature gradually decreased and eventually became similar to room temperature, indicating the end of fermentation. Physical and chemical parameters of the composting process are shown in **Table S1**. At the conclusion of composting, the pH value of the compost was 8.25, meeting the standards for organic fertilizer (pH

value of 5.50 – 8.50). The electrical conductivity (EC) value of the compost on the 30th day was 3.11 mS cm-1, below the threshold of 4 mS cm-1, indicating that it is free from salt contamination(Francou et al., 2005). The carbon-to-nitrogen (C/N) ratio at the end of composting was 13.28. Generally, a C/N ratio below 20 is considered to indicate fully matured compost(Aboutayeb et al., 2025). The above results confirm that the compost has achieved full maturation.

Supplementary Table 1. Physical and chemical changes during composting.

Time (Days)	TOC ($g \cdot kg^{-1}$)	TN ($g \cdot kg^{-1}$)	pH	MC（%）	EC	HS ($g \cdot kg^{-1}$)	HA ($g \cdot kg^{-1}$)	C/N
0	365.76	14.22	6.69	61.06	4.38	112.46	42.86	25.72
2	339.14	11.38	7.46	57.80	3.81	131.73	50.69	29.80
7	320.75	8.55	8.27	54.10	4.08	144.16	62.79	37.51
14	309.44	12.66	8.58	51.30	3.69	136.20	75.24	24.44
21	294.42	18.60	8.47	49.35	3.19	128.33	81.57	15.83
30	282.76	19.30	8.25	48.80	2.55	126.72	91.57	14.65

MC: moisture content. OM: organic matters. TOC: total organic carbon. TN: total nitrogen. C/N: the ratio of total organic carbon to total nitrogen.EC: electrical conductivity. HS: humic substances. HA: humic acid.

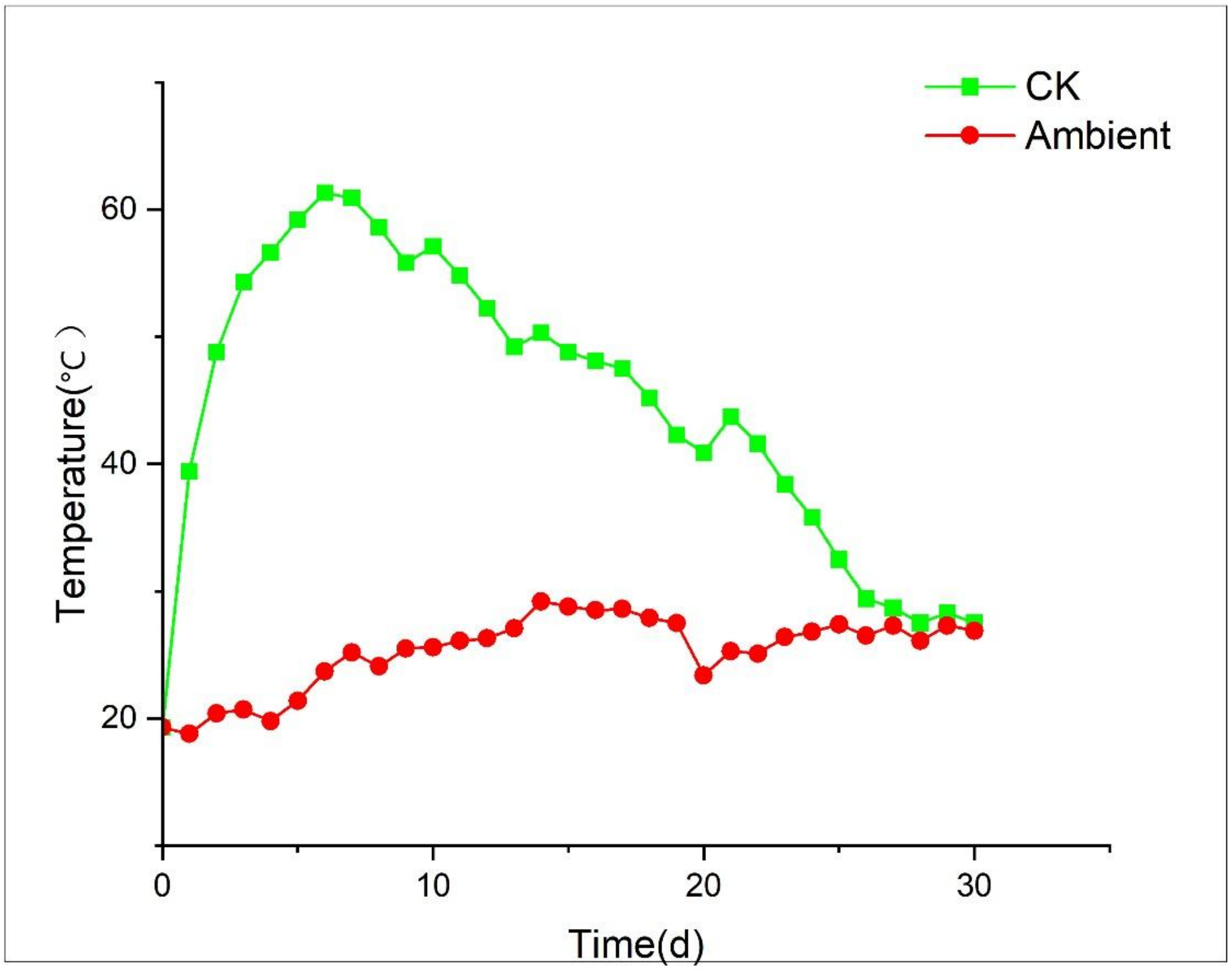

Supplementary Figure 2 Temperature changes during the composting process（CK: temperature changes in the control group; Ambient: environmental temperature changes）

3 Bacterial and Fungal Community Dynamics in Composting Samples Sourced for Strain Screening

Fig.S3a shows the changes in bacterial phylum-level community structure during the composting process. During the mesophilic phase, the dominant phyla were Firmicutes (17.93%) and Proteobacteria (74.89%). During the thermophilic phase, the dominant phyla were Firmicutes (68.07%) and Proteobacteria (11.79%). During the cooling phase, the dominant bacterial phyla were Firmicutes (67.62%), Actinobacteriota (7.21%), and Gemmatimonadota (7.52%). During the cooling phase, the dominant bacterial phyla were Firmicutes (17.36%), Proteobacteria (33.24%), and Actinobacteriota (14.96%). Proteobacteria, Firmicutes, and Bacteroidota play a significant role in the decomposition of cellulose, the mineralization of organic matter, and the cycling of nitrogen, carbon, sulfur, and other substances in composting. Actinobacteria can produce spores to help bacterial strains withstand adverse environments, and they can also positively influence the degradation of cellulose by inducing microorganisms to produce cellulase(Ma et al., 2018; Zhong et al., 2017).

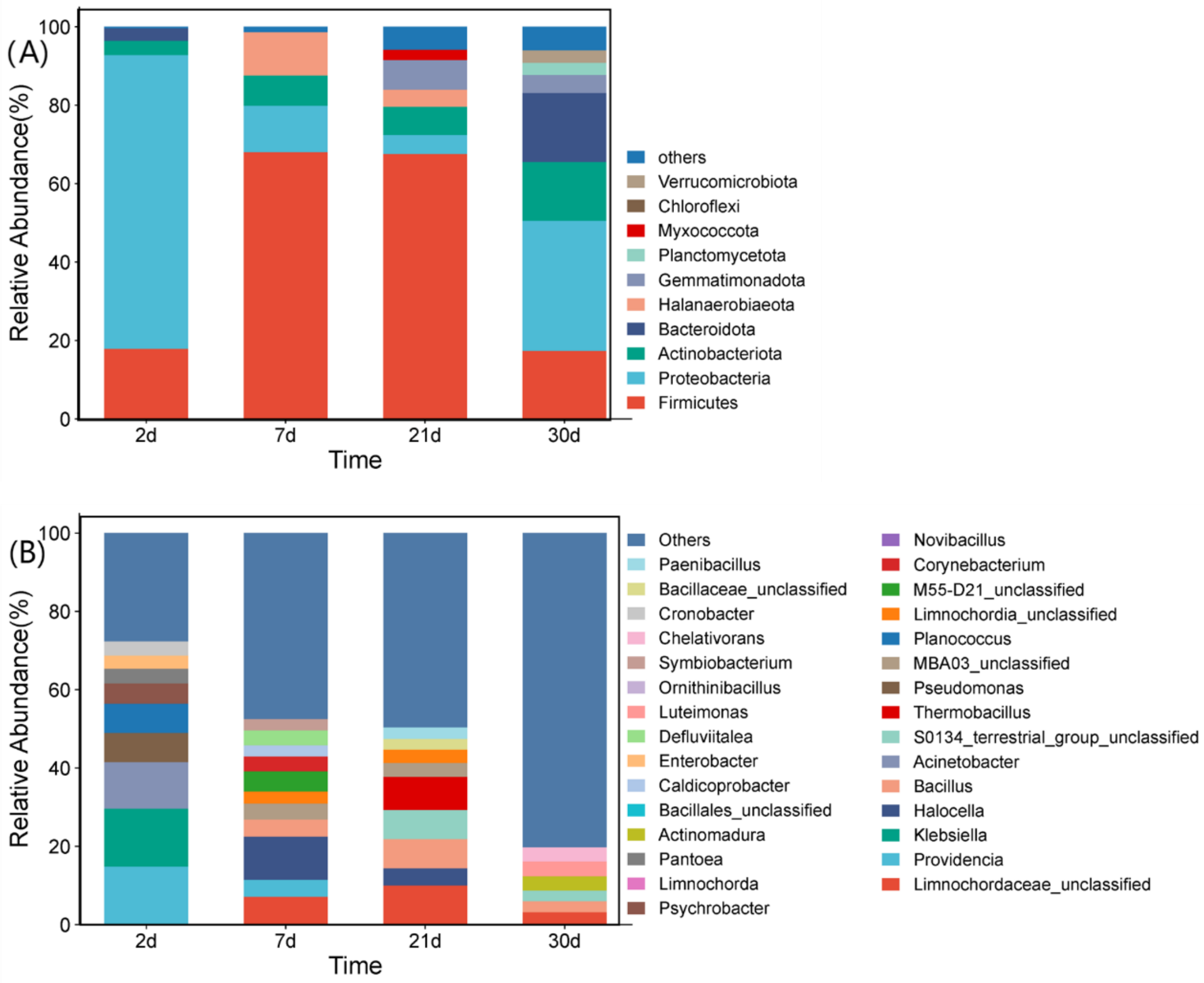

Supplementary Figure 3 Changes in bacterial community diversity during composting: (A) bacterial phylum level（Abundance>2.5%）; (B) bacterial genus level（Abundance>2.5%）

Fig.S3b shows the changes in bacterial genus-level community structure during the composting process. The dominant bacteria during the warming phase were *Providencia* (14.87%), *Klebsiella* (14.79%), *Acinetobacter* (11.90%), *Planococcus* (7.53%), and *Pseudomonas* (7.40%). The dominant bacteria during the thermophilic phase were *Limnochordaceae* (7.16%), *Halocella* (11.07%), and *Bacillus* (4.27%). The dominant bacteria during the cooling phase were *Limnochordaceae* (10.03%), *Halocella* (4.37%), *Bacillus* (7.49%), and *Thermobacillus* (8.41%). The dominant bacteria during the maturation phase were *Limnochordaceae* (3.19%), *Bacillus* (2.14%), *Actinomadura* (3.65%), and *Luteimonas* (3.75%). *Providencia* is a bacterium screened in the laboratory with cellulose-degrading capabilities. *Halocella* is a cellulose-degrading bacterium, and an increase in its abundance leads to an increase in HA/FA(Li et al., 2024). *Klebsiella* can promote nitrogen fixation during the composting process, but it is also an opportunistic pathogen that disappears in the later stages of composting, indicating that the compost product is safe.(Hu et al., 2021) *Bacillus* species exhibit thermotolerance and antifungal properties and can produce various degradative enzymes. The genera *Acinetobacter*, *Lysinibacillus*, *Lactobacillus*, and *Bacillus* are closely associated with organic matter degradation and humification processes(Jiang et al., 2023). The genera *Pseudomonas*, *Paracoccus*, and *Rhodospirillum* are highly associated with organic carbon retention and humus formation(Zhou et al., 2025). *Luteimonas* is a common genus in the composting process and is closely related to the aromatization and humification of soluble organic matter(Fang et al., 2024).

Fig.S4a shows the changes in fungal phylum-level community structure during the composting process. Fungal community structure is simpler and more stable than bacterial structure during composting, but fungi play an important role in the decomposition of organic matter. In the composting system, five fungal phyla were identified, with the primary phyla during composting being *Basidiomycota* (8.29%–90.19%) and *Ascomycota* (7.13%–91.08%). *Ascomycota* and *Basidiomycota*, as dominant fungal phyla, are the main species responsible for the degradation of lignocellulose during composting and play an important role in the decomposition and humification of organic waste(Huang et al., 2022).

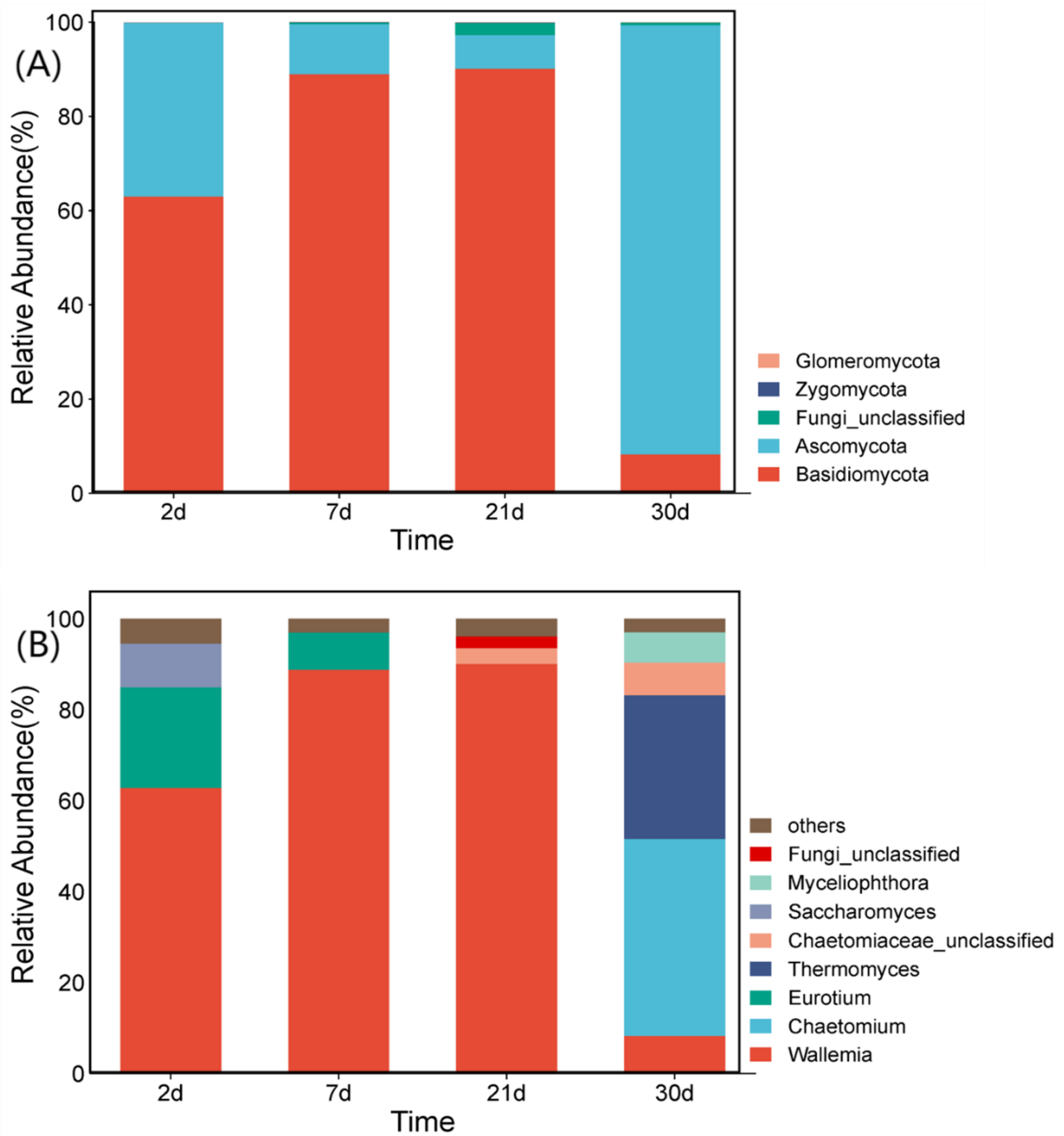

Supplementary Figure 4 Changes in fungal community diversity during composting: (A) fungal phylum level: (B)fungal genus level（Abundance>2.5%）

Fig.S4b shows the changes in fungal genus-level community structure during the composting process. The dominant fungal genera during the initial stage of composting were *Wallemia* (62.82%),

Eurotium (22.11%), and *Saccharomyces* (9.61%). During the thermophilic phase, the dominant fungal genus was *Wallemia* (88.80%). The dominant fungal genus during the cooling phase was *Wallemia* (90.10%). The dominant fungal genera during the maturation phase were *Wallemia* (8.17%), *Chaetomium* (43.34%), and *Thermomyces* (31.65%). Wallemia was widely detected throughout the fermentation process and exhibits salt tolerance(Coleine et al., 2022). *Thermomyces* possesses a large number of genes encoding lignocellulose-degrading enzymes, participating in the degradation of carbohydrates during composting(Liu et al., 2025). *Chaetomium* promotes the degradation of organic matter throughout the composting process, forming small molecules such as amino acids, reducing sugars, and polyphenols, which provide important precursor substances for HS formation(Yan et al., 2023). *Saccharomycaceae* is an important species for lignocellulose degradation, with a relative abundance reaching 50.92%(Verma et al., 2023).

4 Microbial screening results

Supplementary Table 2. Strain screening results.

Serial number	Taxonomy
1	*Enterobacter asburiae*
2	*Providencia vermicola strain ALK059*
3	*Bacillus amyloliquefaciens*
4	*Bacillus subtilis strain D32*
5	*Stenotrophomonas maltophilia strain ISMMS3*
6	*Comamonas terrae strain YSZ*
7	*Cronobacter sakazakii strain fmb11*
8	*Bacillus licheniformis strain EnB-bsys4*
9	*Achromobacter sp. strain BYT-47-2*
10	*Ochrobactrum*
11	*Curtobacterium citreum strain C3*
12	*Bacillus thuringiensis strain BPR162*
13	*Comamonas sp. JJ-Soil-10*
14	*Dyella sp. 24T*
15	*Achromobacter insolitus strain NL40*
16	*Cronobacter sakazakii strain PM467*
17	*Klebsiella sp. HE1*

18	*Achromobacter insolitus strain FA1*
19	*Luteibacter anthropi strain GP5*
20	*Alcanivorax sp. YDC2-H 16S*
21	*Chryseobacterium sp. HNR12*
22	*Halomonas sp. NIMMe9*
23	*Bacillus velezensis strain Y3*
24	*Bacillus subtilis strain FX-8*
25	*Brevundimonas sp. RTB*
26	*Halomonas sp. BJGMM-B48*
27	*Bacillus velezensis*
28	*Bacillus licheniformis strain 51M*
29	*Bacillus licheniformis strain*
30	*Bacillus licheniformis strain QT201*
31	*Lactobacillus fermentum strain 6030*
32	*Lactobacillus salivarius*
33	*Ligilactobacillus sp. strain*
34	*Bacillus cereus strain LXJ76*
35	*Bacillus licheniformis strain WS02*
36	*Delftia tsuruhatensis strain G1-66*
37	*Bacillus licheniformis strain IP_WH4*
38	*Rhodococcus sp. strain IS3-3-6*

39	*Rhodococcus sp.strain S25*
40	*Rhodococcus rhodochrous strain JS-24*
41	*Bacillus tequilensis strain SH41*
42	*Brevibacterium sp. JS5h*
43	*Providencia stuartli*
44	*Aneurinibacillus thermoaerophilus DSM*

5 The phase-specific microbial inoculants

Supplementary Table 3. Composting phase-specific enhanced microbial inoculants

Composting stage	Strain	Source	Type
Mesophilic Phase	*Providencia vermicola strain ALK059*	This study	Bacteria
	Providencia stuartli	This study	Bacteria
	Comamonas terrae strain YSZ	This study	Bacteria
	Klebsiella sp. HE1	This study	Bacteria
	Stenotrophomonas maltophilia strain ISMMS3	This study	Bacteria
	Achromobacter sp. strain BYT-47-2	This study	Bacteria
	Achromobacter insolitus strain FA1	This study	Bacteria
	Halomonas sp. BJGMM-B48	This study	Bacteria
	Brevundimonas sp. RTB	This study	Bacteria
	Brevibacterium sp. JS5h	This study	Bacteria
	Bacillus amyloliquefaciens	This study	Bacteria
	Bacillus subtilis strain D32	This study	Bacteria
	Bacillus licheniformis strain EnB-bsys4	This study	Bacteria
	Acinetobacter calcoaceticus	CGMCC	Bacteria
	Planococcus dechangensis	CGMCC	Bacteria
	Wallemia sebi	CGMCC	Fungus
	Thermomyces lanuginosus	CICC	Fungus
	Chaetomium globosum	CICC	Fungus

	Saccharomyces cerevisiae	CICC	Fungus
Thermophilic Phase	*Providencia vermicola strain ALK059*	This study	Bacteria
	Providencia stuartli	This study	Bacteria
	Halomonas sp. NIMMe9	This study	Bacteria
	Halomonas sp. BJGMM-B48	This study	Bacteria
	Klebsiella sp. HE1	This study	Bacteria
	Curtobacterium citreum strain C3	This study	Bacteria
	Aneurinibacillus thermoaerophilus DSM	This study	Bacteria
	Bacillus licheniformis strain 51M	This study	Bacteria
	Bacillus licheniformis strain	This study	Bacteria
	Bacillus subtilis strain D32	This study	Bacteria
	Bacillus cereus strain LXJ76	This study	Bacteria
	Planococcus dechangensis	CGMCC	Bacteria
	Wallemia sebi	CGMCC	Fungus
	Thermomyces lanuginosus	CICC	Fungus
	Chaetomium globosum	CICC	Fungus
	Saccharomyces cerevisiae	CICC	Fungus
Cooling Phase	*Halomonas sp. NIMMe9*	This study	Bacteria
	Halomonas sp. BJGMM-B48	This study	Bacteria
	Alcanivorax sp. YDC2-H 16S	This study	Bacteria

	Lactobacillus fermentum strain 6030	This study	Bacteria
	Lactobacillus salivarius	This study	Bacteria
	Ligilactobacillus sp. strain	This study	Bacteria
	Rhodococcus sp. strain IS3-3-6	This study	Bacteria
	Rhodococcus rhodochrous strain JS-24	This study	Bacteria
	Bacillus licheniformis strain QT201	This study	Bacteria
	Bacillus velezensis	This study	Bacteria
	Bacillus subtilis strain D32	This study	Bacteria
	Bacillus subtilis strain FX-8	This study	Bacteria
	Wallemia sebi	CGMCC	Fungus
	Thermomyces lanuginosus	CICC	Fungus
	Chaetomium globosum	CICC	Fungus
	Aspergillus niger	CICC	Fungus
	Saccharomyces cerevisiae	CICC	Fungus
Maturation Phase	*Halomonas sp. NIMMe9*	This study	Bacteria
	Halomonas sp. BJGMM-B48	This study	Bacteria
	Luteibacter anthropi strain GP5	This study	Bacteria
	Chryseobacterium sp. HNR12	This study	Bacteria
	Ochrobactrum sp. DS4	This study	Bacteria
	Rhodococcus sp. strain IS3-3-6	This study	Bacteria
	Rhodococcus sp.strain S25	This study	Bacteria

Brevundimonas sp. RTB	This study	Bacteria
Bacillus licheniformis strain 51M	This study	Bacteria
Bacillus licheniformis strain QT201	This study	Bacteria
Bacillus licheniformis strain EnB-bsys4	This study	Bacteria
Bacillus subtilis strain D32	This study	Bacteria
Bacillus tequilensis strain SH41	This study	Bacteria
Wallemia sebi	CGMCC	Fungus
Thermomyces lanuginosus	CICC	Fungus
Aspergillus niger	CICC	Fungus
Saccharomyces cerevisiae	CICC	Fungus

6 The aerobic composting fermentation device

The core of the aerobic composting fermentation device used in this study was a four-unit, double-layer, vacuum-extracted 304 stainless steel fermenter. Each cylindrical tank had an inner diameter of 30 cm, a total height of 80 cm, and an effective volume of 50 L. The fermenter was designed with three vertical zones (upper, middle, and lower). A dedicated feeding port was installed at the top, and a discharge port was set at the bottom to facilitate convenient material loading and unloading. Three sampling ports were arranged vertically on the front side of the tank, corresponding to the upper, middle, and lower zones of the fermenter. On the back side, three temperature sensors were installed vertically to continuously monitor and record the internal temperature changes of the composting material in real time. A porous aeration plate was installed at the bottom of the fermenter. Below this plate, an air inlet channel and a leachate collection and discharge port were provided to separate aeration from leachate treatment. The air inlet was connected to a gas rotameter and a vortex air pump, which enabled forced aeration and oxygen supply inside the tank. During composting, the exhaust gas was directed through a gas outlet at the top of the tank into a dedicated gas collection and purification unit, where it was deodorized before being discharged in compliance with emission standards. A vertical mixing shaft was installed in the center of the tank, with three stainless steel mixing blades fixed onto the shaft. The mixing speed could be flexibly adjusted via a speed control module in the accompanying control cabinet, allowing regular turning of the composting material.

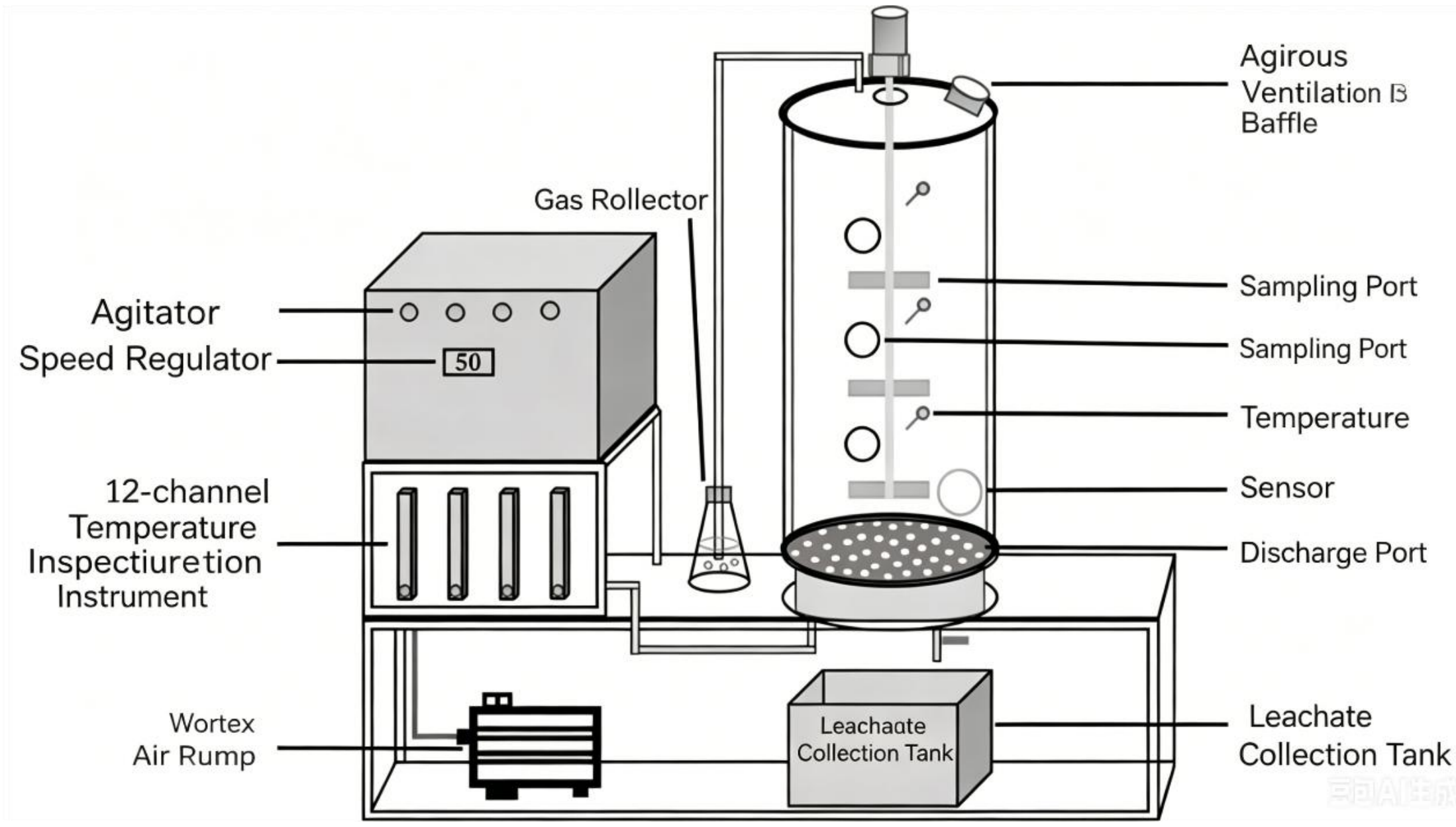

Supplementary Figure 5 Schematic diagram of the aerobic composting fermentation device

References

Aboutayeb R., Fijahi S., Hssaini L., Azim K. (2025) Quality assessment of poultry manure compost: focus on organic amendment and bioremediation roles toward sustainable agriculture. Euro-Mediterranean Journal for Environmental Integration 10:1383–1405. DOI: 10.1007/s41207-024-00698-7.

Coleine C., Stajich J.E., Selbmann L. (2022) Fungi are key players in extreme ecosystems. Trends in Ecology & Evolution 37:517–528. DOI: 10.1016/j.tree.2022.02.002.

Fang C., Su Y., Zhan M.Q., Zhuo Q.T., Yang S.W., Huang G.Q. (2024) Investigating the inhibitory mechanism of methanogenesis during composting under the combined influence of amoxicillin and copper pollution. Journal of Environmental Management 370. DOI: ARTN 123013
10.1016/j.jenvman.2024.123013.

Francou U., Poitrenaud M., Houot S. (2005) Stabilization of organic matter during composting: Influence of process and feedstocks. Compost Science & Utilization 13:72–83.

Hu T., Wang X.J., Zhen L.S., Gu J., Song Z.L., Sun W., Xie J. (2021) Succession of diazotroph community and functional gene response to inoculating swine manure compost with a lignocellulose-degrading consortium. Bioresource Technology 337. DOI: ARTN 125469
10.1016/j.biortech.2021.125469.

Huang Y.H., Chen X.H., Li Q.F., Lu H.X., Mo C.H., Feng N.X., Xiang L., Zhao H.M., Li H., Li Y.W., Cai Q.Y. (2022) Fungal community enhanced humification and influenced by heavy metals in industrial-scale hyperthermophilic composting of municipal sludge. Bioresource Technology 360. DOI: ARTN 127523
10.1016/j.biortech.2022.127523.

Jiang L.Y., Zhao Y.T., Yao Y.Q., Lou J.X., Zhao Y.X., Hu B.L. (2023) Adding siderophores: A new strategy to reduce greenhouse gas emissions in composting. Bioresource Technology 384. DOI: ARTN 129319
10.1016/j.biortech.2023.129319.

Li B.X., Sardar M.F., Zhang X., Ye J., Tian Y.L., Song T.T., Wang X.R., Zhu C.X., Li H.N. (2024) Electrokinetic technology enhanced the control of antibiotic resistance and the quality of swine manure composting. Chemical Engineering Journal 484. DOI: ARTN 149581
10.1016/j.cej.2024.149581.

Liu Q.M., Xie Z.L., Tang S.Y., Xie Q.Q., He X.Y., Li D.J. (2025) Synthetic microbial community enhances lignocellulose degradation during composting by assembling fungal communities. Bioresource Technology 419. DOI: ARTN 132068
10.1016/j.biortech.2025.132068.

Ma S.S., Fang C., Sun X.X., Han L.J., He X.Q., Huang G.Q. (2018) Bacterial community succession during pig manure and wheat straw aerobic composting covered with a semi-permeable membrane under slight positive pressure. Bioresource Technology 259:221–227. DOI: 10.1016/j.biortech.2018.03.054.

Verma S., Awasthi M.K., Liu T., Awasthi S.K., Syed A., Bahkali A.H., Verma M., Zhang Z.Q. (2023) Influence of biochar on succession of fungal communities during food waste composting. Bioresource Technology 385. DOI: ARTN 129437
10.1016/j.biortech.2023.129437.

Yan R.X., Wu H., Yang X.Y., Yang C.Z., Lyu H., Zhang H.W., Li S.Q., Liu T.R., Li R.H., Yao Y.Q. (2023) Soil decreases N2O emission and increases TN content during combined composting of

wheat straw and cow manure by inhibiting denitrification. Chemical Engineering Journal 477. DOI: ARTN 147306
10.1016/j.cej.2023.147306.
Zhao X.Y., He X.S., Xi B.D., Gao R.T., Tan W.B., Zhang H., Li D. (2016) The evolution of water extractable organic matter and its association with microbial community dynamics during municipal solid waste composting. Waste Management 56:79–87. DOI: 10.1016/j.wasman.2016.07.018.
Zhong C.L., Pirch N., Gasser A., Poprawe R., Schleifenbaum J.H. (2017) The Influence of the Powder Stream on High-Deposition-Rate Laser Metal Deposition with Inconel 718. Metals 7. DOI: ARTN 443
10.3390/met7100443.
Zhou L., Wang Z., Li P., Sa R., Wang Z., Wang N., Xie Y., Yang X. (2025) Microbial inoculation influences bacterial and autotrophic community assembly in cow dung-cotton straw composting to promote carbon sequestration and humification. Environmental Technology & Innovation 39. DOI: 10.1016/j.eti.2025.104290.
